# Continental-scale integration of soil metagenomes and organic matter chemistry reveals ubiquitous microbial capacity for chemically-recalcitrant carbon decomposition

**DOI:** 10.1038/s41467-026-71453-5

**Published:** 2026-06-15

**Authors:** Young C. Song, Cheng Shi, Kelly G. Stratton, Christian Ayala-Ortiz, Izabel Stohel, Viviana Freire-Zapata, Malak M. Tfaily, Emiley Eloe-Fadrosh, Emily B. Graham

**Affiliations:** 1 Environmental Molecular Sciences Laboratory, Pacific Northwest National Laboratory, Richland, WA USA; 2 Eawag, Swiss Institute of Aquatic Science and Technology, Dübendorf, Switzerland; 3 Biological Sciences Division, Pacific Northwest National Laboratory, Richland, WA USA; 4 Department of Environmental Science, University of Arizona, Tucson, AZ USA; 5 US Department of Energy Joint Genome Institute, Lawrence Berkeley National Laboratory, Berkeley, CA USA; 6 School of Biological Sciences, Washington State University, Richland, WA USA

**Keywords:** Microbial ecology, Environmental microbiology, Metagenomics

## Abstract

Soil organic matter (SOM) decomposition by microorganisms is a major uncertainty in predicting terrestrial carbon–atmosphere feedbacks, partly because we lack understanding of the microbial diversity involved in depolymerizing different carbon pools across environmental gradients. We address this gap using a continental-scale dataset pairing shotgun metagenomes with high-resolution SOM chemistry, assembling 0.76 Tbp of prokaryotic MAGs (828 genomes) and identifying 66,727 SOM molecules from 47 standardized U.S. soil cores selected using respiration rates from 106 soils. Integrating these datasets reveals widespread microbial potential for depolymerizing chemically-recalcitrant SOM previously considered stable. We uncover complementary metabolic specialization between genera affiliated with two abundant bacterial orders, *Rhizobiales* and *Chthoniobacterales*, and an archaeal order, *Nitrososphaerales*. This metabolic partitioning is consistent across soil depths and activity levels, suggesting coordinated decomposition of complex SOM through distinct but complementary biochemical strategies. The metabolic potential for depolymerization of chemically-recalcitrant compounds is supported by the abundance of these molecules across the soils, as indicated by Fourier-Transform Ion Cyclotron Resonance Mass Spectrometry (FTICR-MS), and by flux balance analysis of metabolic models. Our results show that a substantial portion of ostensibly stable SOM remains vulnerable to microbial decomposition, a mechanism not captured in current Earth System Models.

## Abstract (web summary)

Systematic insights into the diversity of microbes that drive soil organic matter (SOM) decomposition are lacking. In this study, the authors integrate continental-scale soil metagenomics and organic matter chemistry to reveal complementing strategies of microbial SOM degradation across soil depths and respiration levels.

## Introduction

Soil microbial respiration is a critical component of the global carbon cycle, yielding 98 ± 12 Petagram (Pg C/year) of carbon dioxide (CO_2_) into the atmosphere^1^. Most soil carbon is contained within chemically diverse soil organic matter (SOM) pools that, through their interactions with plants, mineral surfaces, water, and other biological and physical soil properties, have the potential to sequester carbon within soils for centuries^2^. Chemically-recalcitrant SOM is typically thought to have long residence times in soils^3,4^, and the widespread potential for its decomposition suggests a possible missing link in the global carbon cycle. Microbial metabolism of SOM proceeds through myriad metabolic processes^5^, yet how interconnected factors and environmental contexts regulate SOM decomposition is largely unknown.

SOM contains a wide variety of organic molecules derived from plant-microbe interactions^5,6^, biomass decomposition, and biochemical transformation into chemically-recalcitrant components^7,8^. These small (<1000 Dalton (Da)) compounds, including amino acids, lipids and organic phosphates, are transformed by soil microorganisms into CO_2_ through respiration^5^. While phylogenetic and metabolic inferences of soil metagenome-assembled genomes (MAGs) have revealed potential microbial mechanisms for transforming various organic molecules^9–11^, robust characterization and confirmation of these pathways have historically depended on isolation and cultivation the organisms represented by MAGs^12^. Alternatively, organic matter characterization approaches using Fourier-Transform Ion Cyclotron Resonance Mass Spectrometry (FTICR-MS) provide molecular-level information about SOM chemistry^5^, which can be integrated with MAGs, potentially enhancing our understanding of SOM transformations while circumventing cultivation bottlenecks.

In this work, we re-evaluate the prevailing view of stabilized SOM as a long-term carbon sink by resolving the microbial and molecular mechanisms that govern SOM decomposition across continental-scale environmental gradients. Given that the predicted residence times of stabilized SOM span decades to centuries, current Earth System Models consider large fractions of SOM as carbon sinks^13^. We add to a growing body of work challenging this notion, while addressing knowledge gaps in the metabolic pathways that regulate decomposition of SOM across environmental gradients, using a unique soil dataset that integrates standardized metagenomics with high-resolution molecular characterization of SOM chemistry collected from surface and subsoils across the Continental United States (CONUS). Through the 1000 Soils Pilot of the Molecular Observation Network (MONet)^14^, we assayed 106 surface and subsoils to uncover complementary roles in continental-scale soil carbon cycling processes conducted by genera within two predominant microbial clades, *Rhizobiales* and *Chthoniobacterales*. Our observations stress the vulnerability of the soil carbon stocks to increased microbial activity under continued environmental change, emphasizing the need for a tractable genetic framework for incorporating microbial mechanisms into Earth System Models.

## Results

We investigated 106 soils that spanned the continental United States, representing 17 of 20 ecoclimatic domains defined by the National Ecological Observatory Network (NEON)^15^. This design enabled us to capture a wide range of spatial variation in soils across the continental United States and is consistent with other published continental-scale works^16,17^. We then assessed variation in microbial communities and SOM chemistry across 47 soils at two depths (surface and subsoil). Soils spanned 37 sites across the CONUS, encompassing most major ecoregions (Supplementary Fig. 1 and Supplementary Data 1). High-resolution mass spectrometry (Fourier-Transform Ion Cyclotron Resonance Mass Spectrometry, FTICR-MS) identified 66,727 distinct SOM molecules (average 7.6 ×10^3^ molecules per sample), while metagenomic sequencing (average 1.1 × 10^8^ reads per sample) yielded 828 metagenome-assembled genomes (MAGs). Taxonomic profiling of the sequenced metagenomic reads indicated that on average 73.1% of them were of microbial origin. Additionally, the assembled MAGs captured 50.6% and 36.5% of the bacterial and archaeal communities at the genus level, respectively.

While we acknowledge that non-prokaryotic organisms such as fungi and viruses have large impacts on biogeochemistry, assembling their metagenomes from soil remains a challenging task for several reasons, including short-read sequencing biases, limited reference databases, and variability in GC content^18–21^. These challenges were evident in our results, as taxonomic classification using Kraken2^22^ revealed that only a small fraction (less than 1%) of the reads from all soil samples were fungal (Supplementary Data 1). Therefore, we focused on bacterial and archaeal communities owing to their substantial roles in soil carbon cycling^23^. Given the above, computational approaches for soil microbiomes allow for deeper investigation into Bacteria and Archaea than other clades^18–21^, and we expect that the results presented here will lay a foundation for investigations into the role of interkingdom interactions on belowground biogeochemistry, as new technological advances in identifying fungi and viruses are available. Detailed procedures of both SOM measurements and MAG assemblies are highlighted in the “Methods” section.

### Prevalence of *Rhizobiales*, *Chthoniobacterales*, and *Nitrososphaerales* across soil samples

**Fig. 1:**
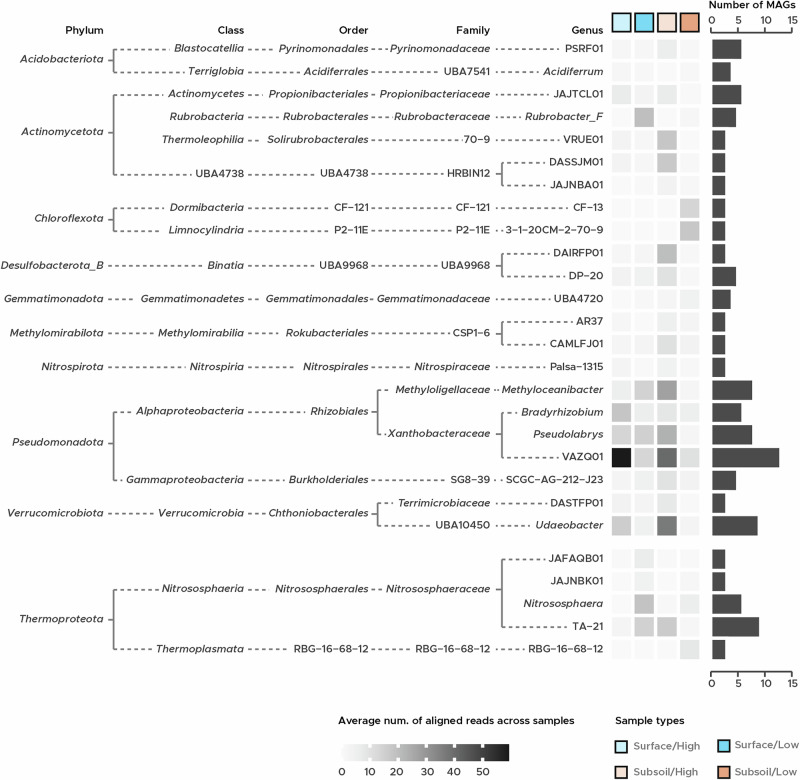
Distribution of bacterial and archaeal MAGs across soil layers and rates of potential microbial respiration. The bar chart shows the number of the dereplicated MAGs that was assigned to each genus-level lineage, while the intensity of the heatmap represents the average numbers of reads from each soil type that were mapped to genomes.

We recovered 828 MAGs, which were dereplicated into 358 genomes. From the dereplicated MAGs, 48 were high quality (completion >90% with contamination <5%) and 310 were medium quality (completion ≥ 50% with contamination of <10%) according to the Minimum Information about Metagenome-Assembled Genome (MIMAG; Supplementary Data 2)^24^. Bacterial MAGs constituted 89.1% of the dereplicated genomes (*n* = 319 MAGs), while the remaining 39 MAGs were archaeal. Phylogenetic inference of the bacterial MAGs (143 from the surface and 176 from the subsoil) revealed that bacterial diversity was dominated by members of *Pseudomonadota* (21.0%; *n* = 67), *Actinomycetota* (23.8%; *n* = 76) and *Acidobacteriota* (17.2%; *n* = 55). Of the archaeal MAGs, 92.3% (*n* = 36) were classified as the phylum *Thermoproteota*, while the remaining three genomes were assigned to the phylum, *Thermoplasmatota*. Nearly 92% of the *Thermoplasmatota* genomes (*n* = 34) were associated with the order *Nitrososphaerales* (Fig. 1).

Examining soil microbial diversity at the order level revealed that *Rhizobiales* (phylum *Pseudomonadota*) and *Chthoniobacterales* (phylum *Verrucomicrobiota*) were the two most prevalent lineages, representing 12.0% (*n* = 43) and 6.1% (*n* = 22) of the dereplicated genomes, respectively (Fig. 1). Relative abundances inferred from read mapping indicated that some genera, such as the candidate group VAZQ01 (order *Rhizobiales*) and the *Udaeobacter* (order *Chthoniobacterales*) were the dominant taxa in high-respiration soils. Both VAZQ01 and *Udaeobacter* were also the two most prevalent genera overall, representing 3.6% (*n* = 13) and 2.5% (*n* = 9) of the total dereplicated MAGs, respectively. Genera within *Rhizobiales*, such as *Bradyrhizobium* and *Methyloceanibacter*, were abundant across all soil types except for low-respiration subsoils.

Unlike *Rhizobiales* and *Chthoniobacterales*, some genera within the phyla *Actinomycetota* and *Chloroflexota* were more prevalent in the subsoils compared to the surface soils. For instance, the candidate genera VRUE01 and DASSJM01, both the members of *Actinomycetota*, had the highest average number of mapped reads in high-respiration subsoils, while the candidate groups CF-13 and 3 − 1 − 20CM − 2 − 70 − 9 affiliated with *Chloroflexota* were the most prevalent genera in low-respiration subsoils. The only exception to this observation was the genus *Rubrobacter_F* within *Actinomycetota*, which was the most prevalent genus in low-respiration surface soils.

Of the genera within the archaeal order *Nitrosophaerales*, the candidate genus TA-21 and *Nitrosphaera* were the most prevalent groups, represented by 9 and 7 dereplicated MAGs, respectively. Both genera had the highest average number of mapped reads in low-respiration surface soils, while TA-21 was also present in high-respiration subsoils.

### Soil chemistry reveals depth-stratified carbon pools with distinct decomposition signatures

**Fig. 2:**
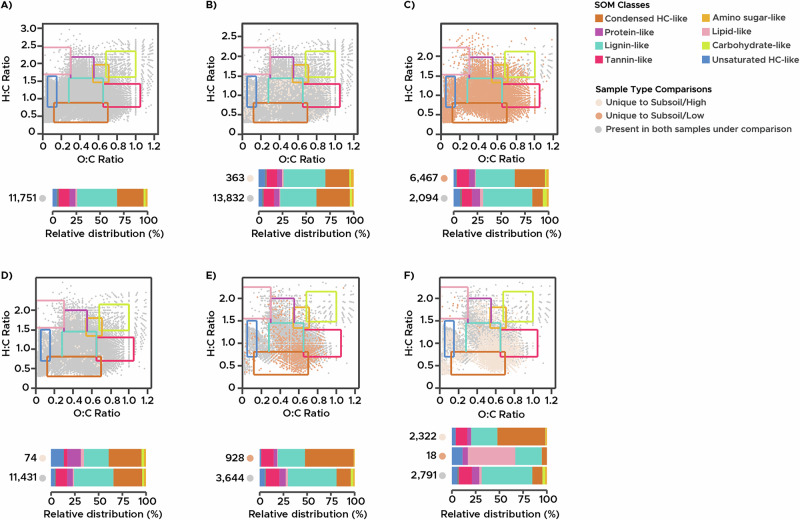
Molecular composition of SOM across soil types. For each comparison, compounds unique to a soil type (unadjusted *p* < 0.005) are represented as dots with colors other than gray, as specified in the legend. The boxes with colored edges on each van Krevelen plot denote SOM classification determined by thresholds of H:C and O:C ratios. The numbers located beside the stacked bar charts indicate the total number of compounds unique to each soil type being compared. The sizes of the colored segments in each bar represent the distribution (as percentages) of SOM classes among the total unique compounds in each soil type. The comparisons between soil samples types are as follows: **A** Surface/High vs. Surface/Low; (**B**) Surface/High vs. Subsoil/High; (**C**) Surface/High vs. Subsoil/Low; (**D**) Surface/Low vs. Subsoil/High; (**E**) Surface/Low vs. Subsoil/Low; and (**F**) Subsoil/High vs. Subsoil/Low.

Pairwise comparisons of SOM molecular composition across depths and potential rates of microbial respiration revealed systematic chemical differentiation with implications for carbon cycling (G-test of uniqueness; Fig. 2 and Supplementary Data 3). While lignin- and condensed hydrocarbon-like compounds were prevalent across all soil types, we observed distinct SOM chemistry between surface and subsoils. For instance, comparison of chemistry in low-respiration subsoils with surface soils (both high- and low-respiration) revealed unique compounds in the subsoils (G-test *p* value < 0.05; Fig. 2C, E; *n* = 8561 and 4572 for C and E, respectively), of which >70% were composed of lignin-, condensed hydrocarbon- and tannin-like compounds.

Similarly, when comparing the chemistry of high-respiration subsoils against high-respiration surface soils, we observed SOM molecules unique to the subsoils, mostly composed (~80%) of lignin-, condensed hydrocarbon-, and tannin-like compounds (Fig. 2B). Comparison between high-respiration subsoils and low-respiration surface soils (Fig. 2D) revealed that while 57% of SOM molecules unique to the subsoils were lignin-, condensed hydrocarbon- and tannin-like compounds, 32.4% were protein- (16.2%) and unsaturated hydrocarbon- (16.2%) like compounds (Fig. 2 and Supplementary Data 3). Between subsoil environments (Fig. 2F), high-respiration subsoils contained 2,322 unique compounds (>75% lignin- and condensed hydrocarbon-like molecules), while low-respiration subsoils had only 18 unique compounds, half of which were lipid-like—potentially reflecting microbial necromass under carbon-limited conditions.

### Microbial potential for depolymerising chemically-recalcitrant SOM is widespread

**Fig. 3:**
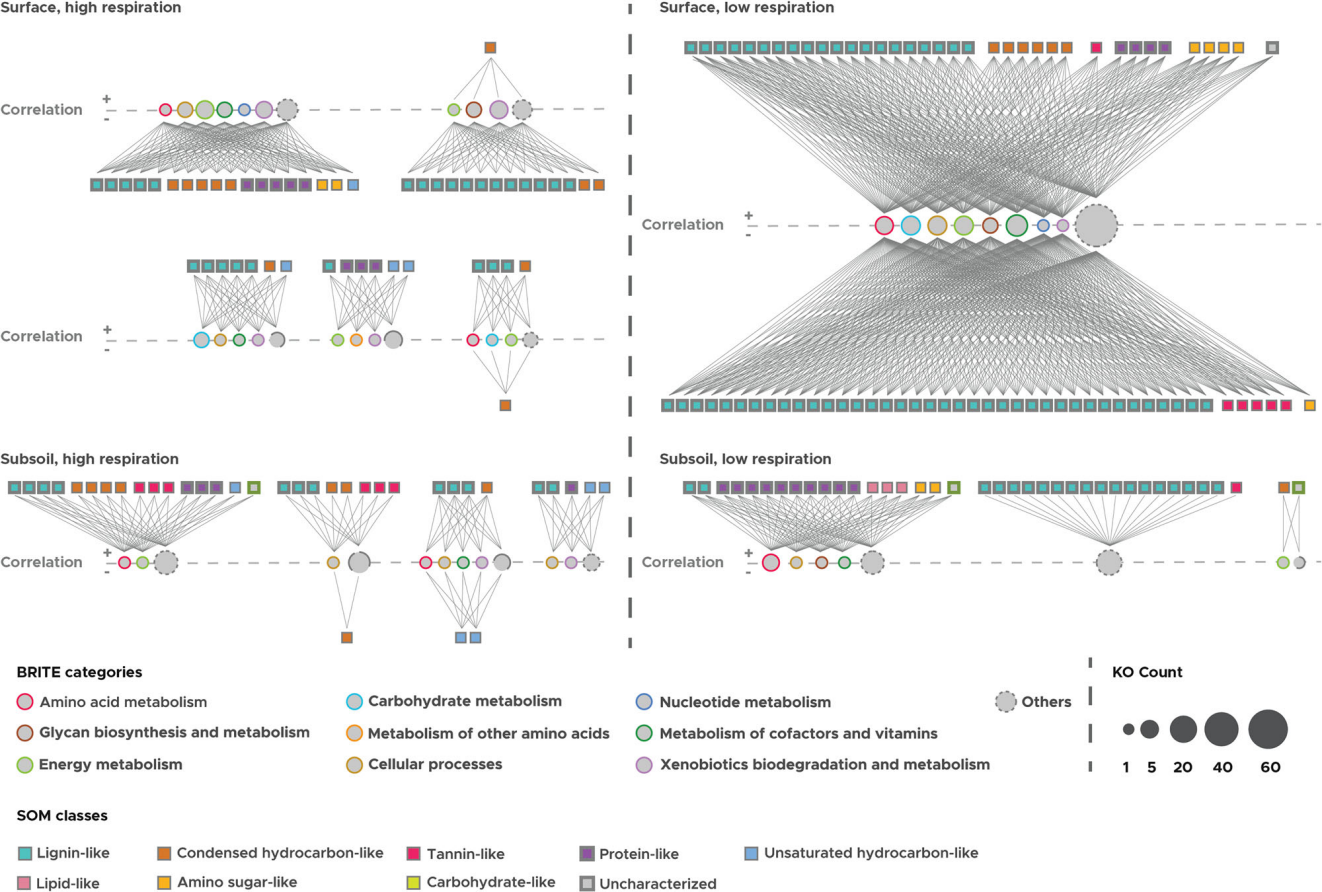
Network modules with significant correlations between SOM and KEGG Orthology (KO)-annotated genes across the soil layers and respiration rates. The shapes of the nodes within each module signify either KO or SOM molecules, with their colors representing KEGG pathway categories for KO or SOM classes, as detailed in the legend. Spearman correlations between SOM and KO were calculated, and a two-sided Spearman’s rank correlation coefficient test was performed. The figures depicts positive and negative Spearman correlations which had FDR-corrected *p* value < 0.01 and |rho| > 0.6, where positive correlations are situated above the dashed line and negative correlations below the dashed line.

Using co-occurrence networks between annotated orthologs from MAGs and SOM molecules acquired from each of the four depth/respiration partitions, we evaluated potential relationships between microorganisms and metabolites. In particular, we identified densely connected modules within a large SOM-KEGG Orthology (KO) network (for all soil layers and potential respiration rates), revealing genes that showed significant relationships with chemically-recalcitrant classes of SOM, such as lignin-, condensed hydrocarbon-, and tannin-like molecules. A case in point is a set of 22 modules recovered in the high-respiration surface soils, with MCODE cluster scores ranging from 2.7 to 19.9 (Fig. 3 and Supplementary Fig. 2). We also recovered a single module from the low-respiration surface soil with a cluster score of 94.2. This module was composed of 189 nodes, 103 of which represented KO-annotated genes that were associated with metabolic pathways such as amino acid metabolism/synthesis, metabolism of C5 sugars, carbon fixation and cellular signaling and processing (Supplementary Data 4). Nearly 75% of the 86 SOM nodes within this module represented lignin-like compounds, while condensed hydrocarbon- and tannin-like compounds were represented by 6 nodes each. In addition, the module recovered from the low-respiration surface soil contained 5 amino-sugar-like compounds, 4 protein-like compounds and one unclassified compound.

The modules identified in high-respiration surface soils revealed the microbial potential for interacting with chemically-recalcitrant carbohydrates, including molecules resembling unsaturated hydrocarbons. Among the 22 modules detected in these soils, 15 contained between 2 and 5 KO-annotated genes linked to pathways such as amino acid catabolism, C5 sugar and starch metabolism, and secondary metabolite biosynthesis (Fig. 3 and Supplementary Fig. 2). Notable genes included *ligXa* and *graA* that are associated with the breakdown of lignin and other chemically-recalcitrant compounds^25,26^. These genes showed positive correlations with various combinations of metabolites, including one or multiple instances of lignin-, condensed hydrocarbon-, protein-, and unsaturated hydrocarbon-like compounds.

At the opposite end of our spectrum of soils, the module describing low-respiration subsoils was consistent with its unique chemistry profile, which included lipid-like compounds that were absent in the surface soils and in high-respiration subsoils (Fig. 3 and Supplementary Data 4). In this module, we observed 306 separate interactions (all represented by edges with correlations of >0.95) between a set of SOM including two lignin-like compounds, 10 protein-like compounds, three lipid-like compounds, two amino-sugar like compounds and an unclassified molecule, and 17 KO-annotated genes (Supplementary Data 4). Five of these genes were associated with amino acid catabolism, while the remaining 12 were linked to cellular functions (e.g., secretion system and biosynthesis of secondary metabolites and cofactors).

**Fig. 4:**
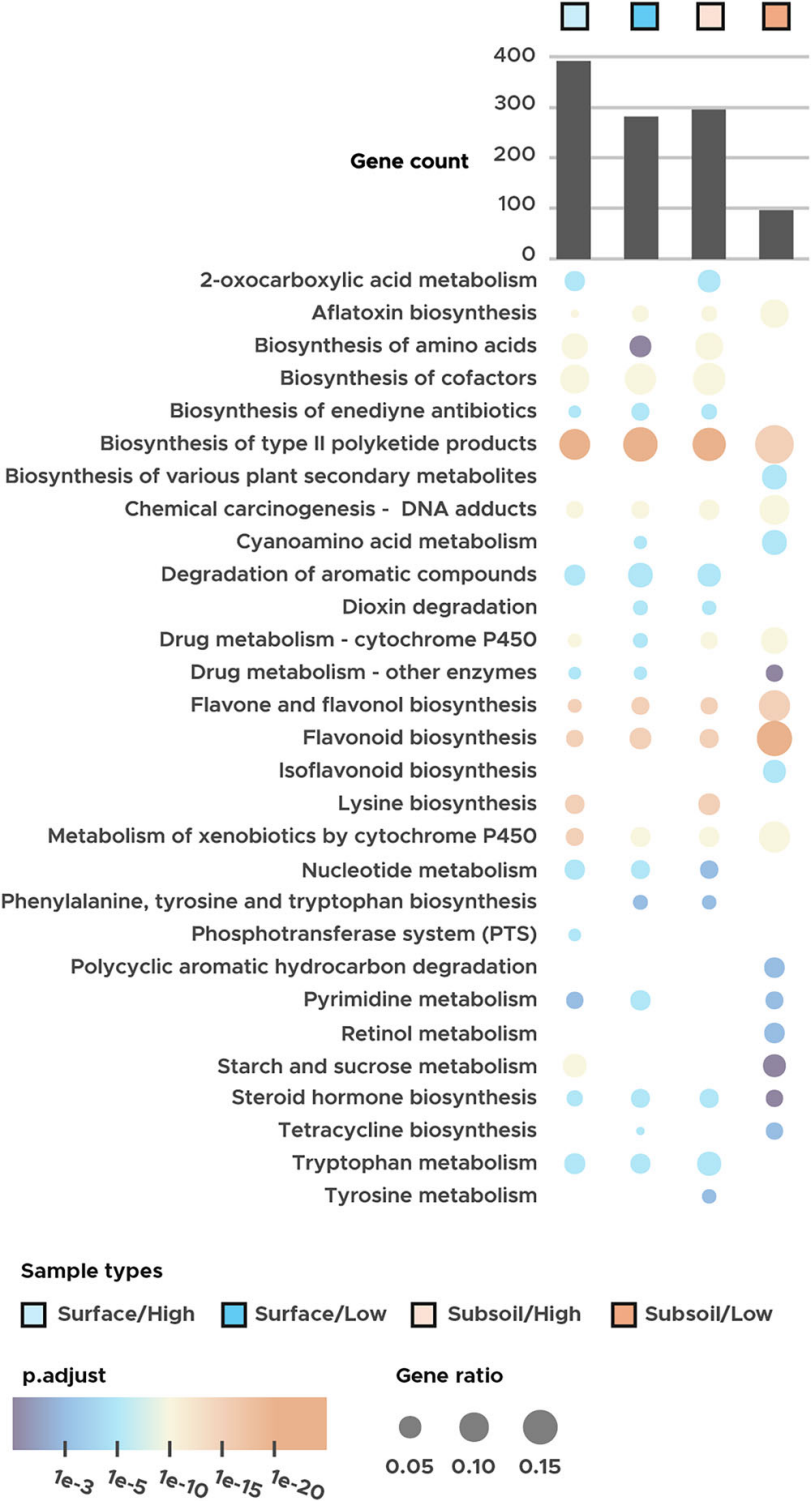
Distribution of BRITE pathway categories based on recruitment of KO-annotated genes mapped to FTICR-MS derived compounds. The histogram depicts the number of genes inferred from SOM profiles of soil samples. The sizes of the bubbles indicate the relative distribution of genes associated with the designated BRITE pathway category, expressed as a proportion of the total gene counts in each sample. The colors of the bubbles represent adjusted p-values, obtained from a two-sided permutation-based modified t-test implemented in the clusterProfiler R package. These p-values were corrected for multiple testing using the Benjamini–Hochberg (BH) false discovery rate method via the enrichKEGG function in clusterProfiler.

Genes in the KEGG database that are associated with metabolites detected by FTICR-MS (see “Methods”) further support the potential for widespread microbial interactions with the chemically-recalcitrant SOM pool and its intermediates^27,28^. For instance, we uncovered metabolites associated with the biosynthesis of cofactors (map01240), which constituted 9.7%, 11.3% and 12.2% of metabolite-associated pathways identified in high- and low-respiration surface soils and in high-respiration subsoil, respectively (Fig. 4). We also detected metabolites affiliated with the metabolism of xenobiotics (map00980) and degradation of aromatic compounds (map01220)––key pathways for processing complex organic polymers. These specialized metabolisms were distributed across all soil environments, constituting between 3.5% to 8.3% of metabolite-associated pathways in all groups of soils (Fig. 4).

### Soil microbial communities universally encode complementary arsenals for depolymerizing chemically-recalcitrant SOM

**Fig. 5:**
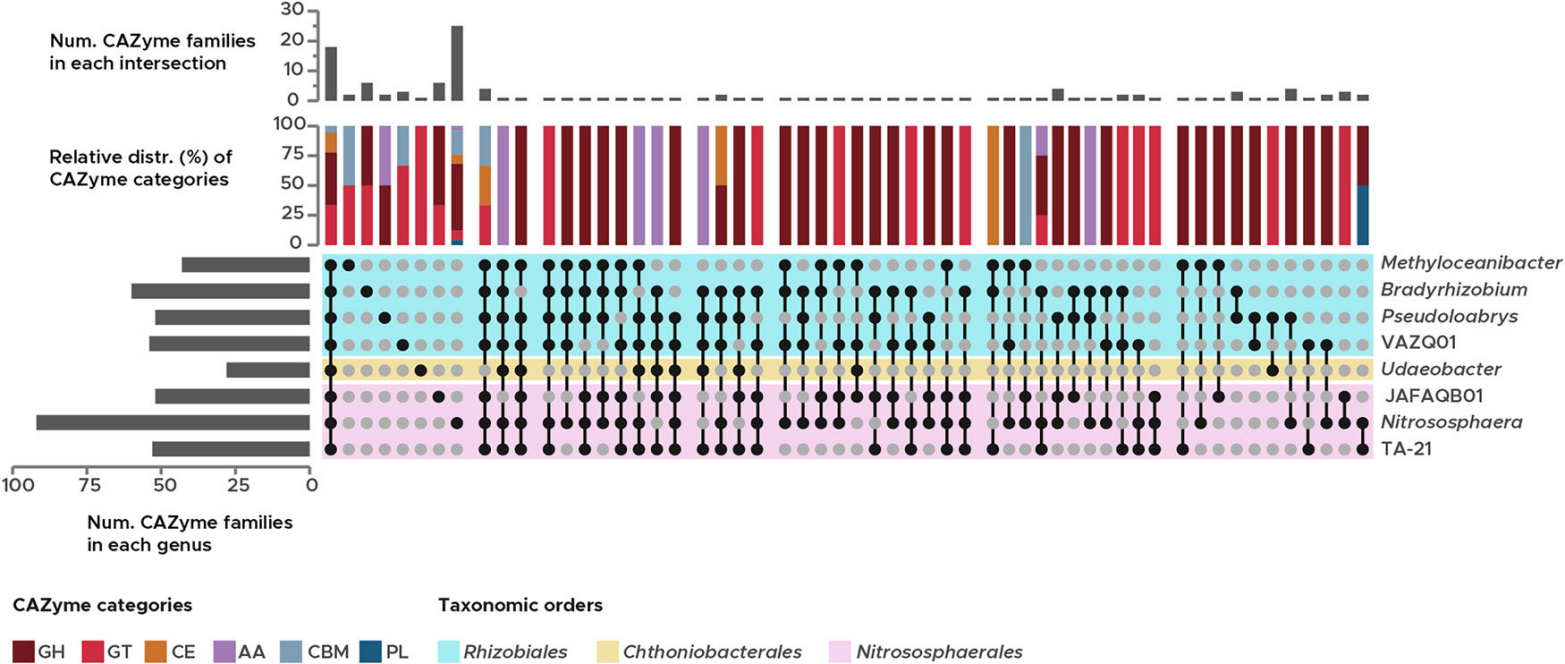
Carbohydrate-active enzymes (CAZymes) detected in selected genera affiliated with *Rhizobiales*, *Chthoniobacterales* and *Nitrososphaerales.* The bar chart on the left illustrates the total number of CAZyme families identified across the selected genera. Meanwhile, the combined dot-and-line plot along with the bar chart at the top displays the count of CAZyme families found in the specified genera or genus. Additionally, the stacked bar chart highlights the proportional distribution of CAZyme categories among these families. The CAZyme categories, as defined in the legend, include GH (glycoside hydrolase), GT (glycosyl transferase), CE (carbohydrate esterase), AA (auxiliary activity), CBM (carbohydrate-binding module), and PL (polysaccharide lyase).

Genomic analysis for carbohydrate-active enzymes (CAZymes)^29^ revealed that the selected genera within the two widespread bacterial orders (*Rhizobiales* and *Chthoniobacterales*) and the archaeal order *Nitrososphaerales* possessed extensive and complementary enzymatic machinery for processing chemically-recalcitrant carbon substrates. Consistent with the prevalence of lignin-, tannin-, and/or hydrocarbon-associated SOM, these genera contained a variety of genes encoding complex carbon depolymerization (Fig. 5). For instance, we detected 92 CAZyme classes in ≥50% of the MAGs assigned to the archaeal genus, *Nitrososphaera*, while recovering 43, 60, 52, and 54 CAZyme classes in ≥50% of the *Methyloceanibacter*, *Bradyrhizobium*, *Pseudolabrys* and VAZQ0, respectively. In addition, 28 CAZyme classes were recovered in ≥ 50% of the genus *Udaeobacter* (Table 1). In all sets glycoside hydrolases (GH) and glycosyltransferases (GT) were the most prevalent CAZyme classes. Unlike the bacterial genera, *Nitrosphaera* and the *Candidatus* TA-21 also possessed polysaccharide lyases (PL).

**Table 1:** Distribution of CAZyme classes detected in selected genera affiliated with *Rhizobiales*, *Chthoniobacterales* and *Nitrososphaerales*

Order	Genus	GH	GT	AA	CBM	CE	PL
*Rhizobiales*	*Methyloceanibacter* (43)	22 (51.2%)	10 (23.2%)	2 (4.6%)	4 (9.3%)	5 (11.6%)	0 (0.0%)
	*Bradyrhizobium* (60)	30 (50.0%)	17 (28.3%)	5 (8.3%)	2 (3.3%)	6 (10.0%)	0 (0.0%)
	*Pseudolabrys* (52)	30 (57.7%)	9 (17.3%)	6 (11.5%)	2 (3.8%)	5 (9.6%)	0 (0.0%)
	VAZQ01 (54)	25 (46.3%)	17 (31.5%)	4 (7.4%)	3 (5.6%)	5 (9.2%)	0 (0.0%)
*Chthoniobacterales*	*Udaeobacter* (28)	12 (42.8%)	8 (28.6%)	4 (14.3%)	1 (3.6%)	3 (10.7%)	0 (0.0%)
*Nitrososphaerales*	JAFAQB01 (52)	28 (53.8%)	16 (30.8%)	1 (1.9%)	3 (5.8%)	4 (7.7%)	0 (0.0%)
	*Nitrososphaera* (92)	49 (53.3%)	21 (22.8%)	5 (5.4%)	8 (8.7%)	7 (7.6%)	2 (2.2%)
	TA-21 (53)	22 (41.5%)	17 (32.1%)	5 (9.4%)	2 (3.8%)	6 (11.3%)	1 (1.9%)

The percentages within the parentheses represent the relative distribution of total classes for each genus, as indicated by the numbers in parentheses beside their names.

A cross-comparison of CAZymes identified in the MAGs from each selected genus further highlighted *Nitrosphaera*’s metabolic potential for degrading a wide variety of chemically-recalcitrant carbohydrates. Of the 92 CAZyme classes recovered from the genomes belonging to this genus, 25 classes were unique to *Nitrosphaera*. Of them, 52% (13) were GH, with the rest classified as GT, auxiliary activity enzymes (AA), carbohydrate esterases (CE), carbohydrate binding molecules (CBM) or PL. The composition of CAZyme classes in the other selected bacterial and archaeal genera were not as diverse as *Nitrososphaera*. For instance, of the 6 enzymes unique to the candidate archaeal genus JAFAQB01, 66.7% (4) were GH, while the remaining two were GT. Similar observations were made in the genera belonging to *Rhizobiales* and *Chthoniobacterales*, where the majority of the CAZymes unique to each of these lineages were dominated by GH, GT and CBM.

While the recovery of fungal MAGs from metagenomic sequencing was limited^18,21^, we uncovered a set of potential fungal pathways for depolymerizing SOM from annotated contigs. In total, we detected 11 CAZymes that were present in fungal contigs in ≥50% of the soils, largely classified as GH, GT, AA or CBM (Supplementary Fig. 3). Of these CAZymes, there was a single AA unique to the high-respiration surface soils and two GT unique to the low-respiration subsoils. We also detected a GH unique to subsoils across both high- and low-respiration.

### Complementary amino acid and inorganic nitrogen acquisition mechanisms are enriched in *Rhizobiales* and *Nitrososphaerales*-associated genera

**Fig. 6:**
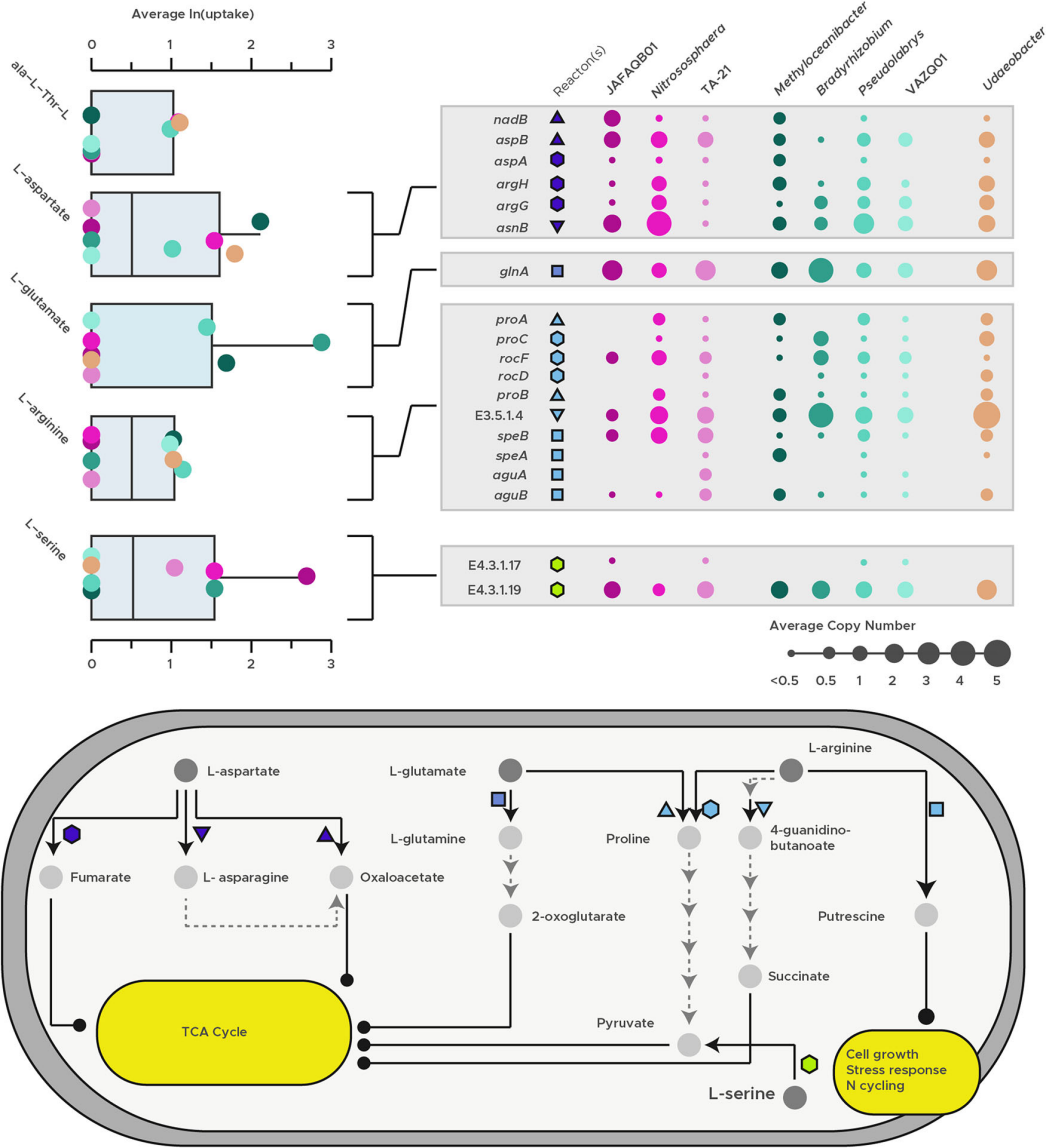
Metabolic potential of amino acid catabolism in the 8 selected bacterial and archaeal genera, based on the FBA and KO annotation of protein-coding genes detected in MAGs. The cell diagram depicts the degradation pathways of the 4 amino acids shown in the boxplot (i.e., L-aspartate, L-glutamate, L-arginine and L-serine). The shapes presented in the cell diagram correspond to the shapes in the left-most column of the bubble diagram. The box plot in the top-left shows the average log-transformed uptake rates of the amino acids for each genus, as determined through FBA. The color coding of the dots/bubbles in both the box and the bubble plots represents bacterial or archaeal genera associated with the three prominent orders, *Rhizobiales*, *Chthoniobacterales* and *Nitrososphaerales*. The left and right edges of the box represent the 25th and 75th percentiles, while the whiskers extend to the most extreme data points within the 1.5 × inter-qualtile range (IQR) of the lower and upper quartiles. The central line in a box (if present) shows the median reaction time. For the box plots associated with ala-L-Thr-L and L-glutamine, the median is equal to the 25th percentile. The numbers of MAGs representing the genera examined via the FBA analysis are as follows: JAFAQB01 (*n* = 3); *Nitrososphaera* (*n* = 3); TA-21 (*n* = 8); *Methyloceanibacter* (*n* = 4); *Bradyrhizobium* (*n* = 3); *Pseudolabrys* ( *n *= 7); VAZQ01 (*n* = 5); and *Udaeobacter* (*n* = 5).

Flux balance analysis (FBA) of the selected genera belonging to *Rhizobiales*, *Chthoniobacterales* and *Nitrososphaerales* revealed that despite the observed metabolic potential for amino acid catabolism in all prokaryotic MAGs, substrate preferences for these amino acids and di-peptide chains appeared to differ by genera (Fig. 6). A closer examination of the selected bacterial and archaeal MAGs revealed a set of genes encoding the transformation of L-aspartate, L-glutamate, L-arginine and L-serine and di-peptide chains to various intermediates of the TCA cycle (Supplementary Data 5). For instance, *Nitrosphaera* and *Udaeobacter* MAGs possessed at least a single copy of most genes encoding transformation of L-aspartate to L-asparagine or oxaloacetate. Meanwhile, the presence of the glutamine synthetase gene (*glnA*), which facilitates the amidation of L-glutamate to synthesize L-glutamine, was more prevalent in *Bradyrhizobium* compared to other genera. FBA results were supported by the distribution of KO-annotated genes, which showed metabolic potential for transforming both L-aspartate and L-glutamate in *Methyloceanibacter* and *Pseudolabrys*. The annotation profiles for the bacterial and archaeal genera aligned with the FBA, which indicated a high average uptake rate of L-aspartate and/or L-glutamate for for *Nitrosphaera*, *Udaeobacter* and most genera in *Rhizobiales*.

The transformation of L-arginine was notably prevalent among *Methyloceanibacter*, *Pseudolabris*, candidate genus VAZQ01, and *Udaeobacter*, with these four bacterial genera exhibiting a higher average uptake of L-arginine compared to other lineages under study (Fig. 6). Interestingly, MAGs associated with *Bradyrhizobium* and *Udaeobacter* displayed a high average copy number of the gene encoding aliphatic amidase (*amiE*), an enzyme that catalyzes the hydrolysis of short-chain amides generated from the activity of arginine monooxygenase^30^. However, the average L-arginine uptake in *Bradyrhizobium* was close to zero, unlike in *Udaeobacter*. Additionally, most *Bradyrhizobium* MAGs lacked genes associated with the conversion of L-arginine to putrescine (e.g., *speB*, *speA*, *aguA*, and *aguB*), distinguishing them from other *Rhizobiales*-associated genera. Similarly, the same genes were absent in JAFAQB01 and *Nitrososphaera*. These archaeal genera, along with TA-21, exhibited an almost negligible average L-arginine uptake rate. All bacterial and archaeal MAGs contained at least one copy of *ilvA*, which encodes L-serine/L-threonine dehydratase, an enzyme responsible for converting serine into pyruvate. Among these genera, only the three archaeal genera and *Bradyrhizobium* demonstrated a comparable average L-serine uptake rate. These patterns suggest complementary amino acid utilization strategies between bacterial and archaeal lineages, whereby L-arginine catabolism is concentrated in genera such as *Methyloceanibacter*, *Pseudolabrys*, candidate genus VAZQ01, and *Udaeobacter*, while archaeal taxa and *Bradyrhizobium* rely more consistently on L-serine, L-aspartate and L-glutamate rather than L-arginine-based pathways.

In addition to complementary amino acid catabolism, bacterial and archaeal lineages also displayed partitioned pathways for inorganic nitrogen transformations, with *Nitrosphaerales* specializing in ammonia oxidation and key bacterial genera encoding dissimilatory nitrate reduction. A nearly complete set of genes involved in ammonia oxidation to nitric oxide (NO), including *amoCAB* and *nirK*, was found across all three *Nitrosphaerales* genera (Supplementary Data 5). However, the *hao* gene, which encodes hydroxylamine dehydrogenase, was notably absent in all *Nitrosphaerales* MAGs. In contrast, ammonia oxidation genes were entirely absent from *Rhizobiales* and *Chthoniobacterales* MAGs. Instead, a partial set of genes involved in dissimilatory nitrate reduction, such as *narGHI*, *napAB*, *nirBD*, and *nrfAH*, were detected, particularly in *Methyloceanibacter* and *Udaeobacter* (Supplementary Data 5).

### Lipid metabolism reveals contrasting survival strategies under carbon limitation

The distribution of SOM molecular classes across soils revealed that while microbial potential for depolymerizing chemically-recalcitrant organic matter appeared to be widespread in surface soils and in high-respiration subsoils, biomass-synthesis pathways tended to dominate low-respiration subsoils. A complete or near-complete set of lipopolysaccharide biosynthesis genes (e.g., *lpxD*, *lpxL*, and *gmhC*) were identified in MAGs across nearly all bacterial and archaeal genera studied, with the exception of *Bradyrhizobium* (Supplementary Data 6). Notably, these genes were present in ≥ 50% of the MAGs affiliated with *Nitrososphaera* and *Udaeobacter*, highlighting fundamentally different strategies in these microbial lineages for surviving carbon limitation. For example, *Nitrososphaera* and *Udaeobacter* prioritize cellular persistence via enhanced cell-envelope production and maintenance, while other taxa may depend more on aggressive breakdown of complex organic substrates to meet their carbon and energy demands^31,32^.

We also investigated the occurrence of lipid biosynthesis genes in MAGs associated with two candidate genera, CF-13 and 3-1-20CM-2-70-9, both classified under the phylum *Chloroflexota* and the most prevalent taxa in low-respiration subsoils (Fig. 1). Compared to MAGs of dominant bacterial and archaeal lineages, the genomes of CF-13 and 3-1-20CM-2-70-9 exhibited lower CheckM completion rates (between 50% and 80%). Only a partial set of lipopolysaccharide synthesis genes was detected in a small fraction of these MAGs, consistent with their relatively lower genome completion rates relative to those of more prevalent bacterial and archaeal lineages. These patterns suggest that the dominant *Chloroflexota* taxa in low-respiration subsoils may rely less on canonical lipopolysaccharide-based cell envelope structures than other lineages, although more complete genomes will be required to confirm this inference.

## Discussion

Our study provides evidence that soil microbiomes across the continental United States possess genetic potential associated with the depolymerization of compounds traditionally considered to be chemically stable, including complementary metabolic specialization among abundant bacterial (*Rhizobiales*, *Chthoniobacterales*) and archaeal (*Nitrososphaerales*) lineages. While it is well established that soil microorganisms contribute to the degradation of organic substrates over time, our work advances this understanding by coupling high‑resolution, molecular‑level characterization of SOM chemistry with an in-depth analysis of prokaryotic metagenome-assembled genomes. This widespread microbial capacity for chemically-recalcitrant SOM turnover underscores the need for further investigations into the specific enzymatic pathways (e.g., CT-depolymerization, C_15_ metabolism and phenolic metabolism^33^) that appear to support the breakdown of chemically-recalcitrant compounds in most soils. By integrating metagenomes with molecular-level SOM measurements, we identified genera within dominant bacterial and archaeal orders—*Rhizobiales*, *Chthoniobacterales* and *Nitrosospaherales*—that use complementary enzymatic strategies to process chemically-complex carbon across diverse soils. Our multi-omic integration also shows that these strategies shift with depth, from depolymerization-dominated metabolisms in surface and high-respiration subsoils to biomass-synthesis–oriented metabolisms in low-respiration subsoils. These findings suggest that slow carbon pools in current models may be more vulnerable to decomposition than previously thought, and that this vulnerability is modulated by depth-dependent variation in microbial metabolism, exposing a critical gap in carbon-climate feedback predictions.

It is important to note that SOM degradation not only depends on the chemical composition of SOM, but also on factors that control how easily microorganisms can encounter and use that material^16,17,34,35^. The rate at which SOM turns over is governed both by the chemical properties of carbon inputs and by physical and spatial constraints that limit contact between microorganisms and organic substrates. Organic compounds that are chemically bioavailable could be protected from microbial decomposition by physical barriers that restrict access or create micro‑environmental constraints on decomposer activity and movement—for example, protection within soil aggregates or nanopores, or isolation in ice or anaerobic microsites^36,37^. These abiotic factors themselves could also be influenced by chemistry (e.g., chemical binding). Although we focus on dissolved SOM, particulate organic carbon also plays key roles in nutrient cycling, carbon storage, and the formation of stable organic matter fractions that underpin soil health and ecosystem sustainability^38,39^. Together, these perspectives emphasize that accurate representation of soil carbon dynamics will require coupling molecular‑level descriptions of SOM chemistry with explicit consideration of microbial physiology and the spatially heterogeneous soil environment.

Integrating chemical profiling of SOM and functional annotation of soil MAGs showcases widespread microbial potential for degrading lignin-, condensed hydrocarbon-, and tannin-like molecules that are typically thought to have long residence times in soils (Figs. 3 and 4)^40^. These dynamics may arise from microbial interactions with plant residues and their decomposition products, which are shaped by the molecular composition and spatial arrangement of SOM, as well as the functional potential of the co-located microbiome^40–42^. The prevalence of lignin- and condensed hydrocarbon-like molecules across soil depths in particular, regardless of microbial activity level, and their association with genes encoding SOM-degrading enzymes suggest current soil carbon stocks may be vulnerable to microbial activity in response to changing environmental conditions (Figs. 2, 3, and 4)^43,44^. The apparent persistence of the soil carbon pool therefore likely reflects limits on microbial access or activity, and the shifts in factors such as temperature, moisture, or redox conditions could relax these constraints and stimulate additional decomposition of existing soil carbon stocks. Identifying genes, such as *ligXa* and *graA*, directly linked to chemically-recalcitrant carbon processing highlights potential microbial mechanisms in decomposing chemically-recalcitrant carbohydrates, such as lignin to simpler molecules^25,26,45^. Additionally, genes for polyketide synthesis and other secondary metabolites may contribute to SOM depolymerization through mechanisms like metal mobilization^46,47^ and the activation of quorum sensing activation^48^. The detection of complex networks in the low-respiration surface soils, together with prevalence of *Methyloceanibacter*, *Pseudolabrys*, and *Nitrososphaera* at the same soil depth and respiration level further underscores the increased reliance of these microorganisms on accessible carbohydrates during periods of low microbial activity (Figs. 1 and 3). While these bacterial and archaeal lineages are primarily known for their C1 and nitrogen metabolism (e.g., nitrogen fixation or nitrate reduction), the presence of CAZymes for depolymerizing complex carbon polymers in some members of these clades has also been previously described^49–53^. Together, this suggests that under low microbial activity, communities in surface soils are supported by taxa such as *Methyloceanibacter* and *Pseudolabrys*, which encode diverse CAZymes and central carbohydrate pathways alongside L-aspartate-, L-glutamate-, and L-arginine-catabolic routes, and by *Nitrososphaera* with its capacity for ammonia oxidation, enabling continued turnover of accessible complex SOM even when bulk respiration is low.

The widespread presence of *Rhizobiales*, *Chthoniobacterales*, and *Nitrososphaerales* across all soils, combined with the abundance of CAZymes in the MAGs associated with genera within these orders, suggests mechanisms for the depolymerization of SOM during both high and low microbial activity in surface and subsurface soils (Fig. 5). Notably, the prevalence of GH and GT highlights their ability to interact with glycans, monosaccharides, and polysaccharides, in particular, and to construct extracellular structures that support adaptation to varying levels of carbon availability^54,55^. Additionally, the detection of carbohydrate-binding modules (CBM) unique to genera within *Rhizobiales*, such as *Methyloceanibacter* and VAZQ01, further emphasizes the potential of these microorganisms to break down chemically-recalcitrant compounds, particularly under low microbial activities.

*Nitrosphaera* in particular contained a distinct set of CAZymes, exhibiting greater diversity compared to those found in other bacterial or archaeal MAGs (Fig. 5). While members of this genus have traditionally been identified as ammonia-oxidizing chemolithotrophs, recent studies indicate metabolic versatility within certain *Nitrosphaera* sub-clades^49^. In support of this emerging idea, we detected both intracellular and extracellular CAZymes, such as CBM and GH, alongside genes involved in ammonia oxidation (Table 1 and Supplementary Data 5). Additional evidence for metabolic diversity in *Nitrosophaera* includes the presence of amino acid catabolic pathways, particularly for L-aspartate and L-serine (Fig. 6). Given the prevalence of *Nitrosophaera* in low-respiration surface soils, as well as in subsoils with limited respiration, we hypothesize that these Archaea are equipped with mechanisms utilize amino acids as a source of nitrogen required to depolymerize complex carbon compounds, enabling them to persist under energy- and carbon-limited conditions.

In *Nitrosphaera* and other genera within *Nitrosphaerales*, pathways involving amino acids appear to be coupled with ammonia oxidation, given that none of the MAGs belonging to these groups possessed the *hao* gene, which is thought to be an essential component of ammonia oxidation^56–58^. The absence of *hao* may reflect sequencing limitations or suggest the involvement of uncharacterized Cu-protein complexes in ammonia oxidation, a mechanism observed in other ammonia-oxidizing Archaea (Supplementary Data 9)^57^. Ammonia that is oxidized by *Nitrososphaerales* could be generated from catabolism of available amino acids in soil. These findings underscore the role of *Nitrososphaerales* in linking soil carbon and nitrogen cycles and highlight the need for further investigation into their genetic machinery for ammonia oxidation.

Amino acid and inorganic nitrogen acquisition mechanisms were also enriched in *Rhizobiales* and *Chthoniobacterales*, as observed in flux balance analyses of the MAGs, indicating a vital role of these pathways in nitrogen acquisition from SOM and energy generation in *Rhizobiales* and *Chthoniobacterales* in particular (Fig. 6 and Supplementary Fig. 2)^59,60^. In *Rhizobiales*- and *Chthoniobacterales*-affiliated genera, particularly in *Methyloceanibacter* and *Udaeobacter*, intermediates generated through amino acid catabolism (e.g., pyruvate, NADH, and FADH_2_) could serve as sources of reducing power to drive dissimilatory nitrate reduction, which was also detected in these lineages (Supplementary Data 5). Therefore, the degradation of amino acids like L-arginine, observed in *Methyloceanibacter* and *Udaeobacter*, along with their prevalence in soils with low microbial activity, indicates that amino acid catabolism is crucial for maintaining energy balance and supporting nitrogen biosynthesis required for fundamental cellular functions (Figs.1 and 6)^59–62^.

The broad spectrum of CAZyme classes identified in *Rhizobiales*-affiliated genera versus other MAGs highlights a putative advantage for them to break down chemically-recalcitrant SOM in soils with high microbial activity. This is especially evident for *Bradyrhizobium*, *Pseudolabrys*, and VAZQ01, which were prevalent in both surface and subsoils with elevated respiration rates (Figs. 1 and 5). While the potential to decompose complex carbohydrates has been documented for both *Bradyrhizobium* and *Pseudolabrys*, the metabolic characteristics of VAZQ01 remain poorly understood^63,64^. Although the *Chthoniobacterales*-affiliated lineage *Udaeobacter* was also present in both surface and subsoils with high respiration, the limited set of CAZymes detected in its MAGs indicates a dependence on mono- and polysaccharides for carbon and energy metabolism, further reinforcing its auxotrophy for various amino acids. (Figs. 5 and 6^65^). The amino acid catabolism observed in *Udaeobacter*, combined with its previously identified ability to sustain the respiratory chain using atmospheric H_2_ under nutrient-deficient conditions, aligns with its prevalence in high-respiration subsoils, where carbon and nitrogen sources are scarce and competition for these resources is intense (Fig. 1^66,67^). These findings suggest contrasting carbon usage strategies in *Rhizobiales* and *Chthoniobacterales*, with *Rhizobiales* utilizing a broader range of carbohydrate polymers and amino acids as sources of both carbon and nitrogen, whereas *Chthoniobacterales* relies on scavenging amino acids, likely derived as byproducts of microbial activities in soil, highlighting how taxon-specific metabolic traits can shape nutrient cycling and microbial interactions in terrestrial ecosystems.

Lastly, the bioavailability of soil lipids has been widely debated, because they may dually serve as a high-energy substrate and as a reservoir of sequestered carbon derived from microbial necromass^68–70^. We found a diversity of lipid-like molecules in low-respiration subsoils that may represent sequestered plant and/or microbial residues from prior periods of higher metabolic activity^71,72^ (Fig. 2). In subsoils, nutrient limitations and/or mineral protection likely promote the disproportionate accumulation of lipid-like molecules, contributing significantly to the stable fraction of SOM. While lipid persistence has been linked to soil carbon stabilization, the universality of soil microbial potential for lipid metabolism – even in soils with low rates of measured metabolic activity – suggests that they may become susceptible to decomposition as environmental conditions change. Notably, interactions between lipid-like molecules and microbial genes in low-respiration soils also indicate that lipid-like necromass components may be vulnerable to microbial decomposition during periods of reduced carbon availability and respiration rates^72,73^ (Fig. 3). Thus, we highlight potential lipid turnover as a key consideration in soil carbon dynamics^51^.

Strong network correlations between lipid-like compounds and microbial secretion systems, cofactors, and secondary metabolites in low-respiration subsoils may also elucidate strategies for microbial adaptation to carbon-limited environments (Figs. 3 and 4; Supplementary Data 6). Secretion systems enable microbial interactions by exporting compounds to facilitate nutrient acquisition, particularly labile substrates^74,75^, while cofactors (e.g., molybdopterin) and secondary metabolites support energy metabolism, redox reactions, and oxidative damage mitigation under stress^76,77^. The functional annotations of the MAGs linked to *Rhizobiales*, *Chthoniobacterales*, and *Nitrososphaerales* suggest that these microbes may possess the ability to enhance lipopolysaccharide (LPS) synthesis, thereby stabilizing their cell envelope and boosting their resistance to environmental stress^78,79^. These characteristics were notably prevalent in *Udaeobacter* and *Nitrososphaera* (Supplementary Data 6). Gram-negative Bacteria, including *Udaeobacter* and *Nitrososphaera*, contribute significantly to organic carbon storage through the chemical persistence of LPS, particularly its chemically-recalcitrant lipid A component^80,81^. On cell death, released LPS acts as a binding agent between soil particles, enhancing soil stability and water retention^82^. These adaptations highlight how microbial communities persist and exploit resources under carbon deficiency while shaping key soil ecosystem processes like organic carbon cycling and storage (Figs. 1 and 2).

While we focus on bacterial and archaeal regulation of SOM cycling, our study also highlights the need for investigating abiotic processes and interactions with fungi and viruses, which are harder to disentangle using current metagenomic methods^18–21^. Assembling and annotating fungal metagenomes presents clear challenges, as evidenced by the smaller fraction of reads and the relatively low number of assembled contigs compared to those of bacterial or archaeal origin; however, the annotation of these contigs revealed the potential role of soil fungal communities in contributing to soil carbon flux. We also note that FBA-derived metabolic inferences assume microorganisms operate in a steady state, and the incomplete recovery of MAGs may limit interpretation, warranting validation of these inferred traits using transcriptomics and/or proteomics; in particular, validation through cultivation methods and emerging computational tools for co-assemblies^83,84^ would enhance the robustness of the findings. The recovery of 828 MAGs—though indicative of substantial prokaryotic diversity—may be biased toward ubiquitous, abundant taxa favored by continental-scale sampling across geographic gradients, and site-specific specialists and rare taxa involved in specialized carbon transformations^85,86^ are likely underrepresented, suggesting that the results primarily reflect the “core” microbial carbon cycling machinery rather than the full functional diversity present in soil communities. Still, the multi-omics integration in this study have facilitated the recovery of bacterial and archaeal lineages with unique yet complementary carbon sequestration strategies.

Our study provides a comprehensive, genome-resolved view of microbial decomposition potential across diverse soil environments, illuminating the metabolic underpinnings of carbon turnover at continental scale. By integrating thousands of chemicall- distinct SOM molecules with hundreds of metagenome-assembled genomes, we uncover widespread microbial capacities to degrade SOM long thought to be chemically-resistant to decay. This work helps fill a critical knowledge gap in linking microbial taxonomy and metabolism to specific carbon pools, offering a mechanistic foundation for improving predictions of soil carbon dynamics under shifting environmental conditions. As global change accelerates, incorporating these microbial processes into Earth system models will be essential to more accurately project the fate of soil carbon reservoirs.

## Methods

### Data Collection

Through the 1000 Soils Pilot of the Molecular Observation Network (MONet)^14^, 109 soil samples were collected from 60 sites across the CONUS. Full methodological details are available in refs. (^14,87^), and at https://github.com/EMSL-MONet/MONet-Protocols-. With a goal to investigate the microbial pathways that underscore variation in microbial activity across geography and soil depth profiles, we selected the subset of 47 soil samples, which exhibited the highest and lowest (30%) rates of respiration within either surface (0-10 cm) or subsoils (20-30 cm) for analysis. These soil samples spanned 37 geographical locations (Table 1) and were divided into four groups based on depth and respiration: surface-high (*n* = 14), surface-low (*n* = 12), suboil-high (*n* = 11), and subsoil-low (*n* = 10).

### Fourier transform ion cyclotron resonance mass spectrometry (FTICR-MS)

Water-soluble SOM composition was analyzed by Fourier Transform Ion Cyclotron Resonance Mass Spectrometry (FTICR-MS)^87,88^. Briefly, 6 g of dried soil was resuspended in 30 mL DI water in triplicate, and shaken for 2 h at 800 rpm. The samples were then centrifuged at 6000 rpm for 8 min and 5 mL of supernatant was transferred for solid phase extraction (SPE) with Agilent Bond Elut PPL cartridges^88^ on Gilson ASPEC® SPE system. We used a Bruker 7 Tesla scimaX FTICR-MS at the Environmental Molecular Sciences Laboratory (EMSL) in Richland, WA to analyze the extracted SOM, with a negative electrospray ionization mode and ion accumulation time of 0.01 or 0.025 s. The mass accuracy was below 1 ppm, as confirmed by the Suwannee River Fulvic Acid (SRFA) samples (Supplementary Information). We used CoreMS (v. 2.0.0)^89^ to process the raw FTICR-MS data, with noise thresholding of 10 (*noise_threshold_log_nsigma*) and minimum peak prominence of 0.1 (*peak_min_prominence_percent*). Mass calibration was performed by setting a threshold that uses the most calibration points (usually >200). Molecular formulae were annotated by both accurate mass and isotopologues, with a confidence score calculated for each formula. We then filtered the assigned peaks by *m/z* between 200 and 1000, present in at least 2 out of 3 replicates, not present in two or more lab blanks, and with formulae confidence scores (combines *m/z* error and isotopic pattern) above 0.5^89^.

### Distribution and uniqueness of SOM across soil samples

Using a customiz Python script, we classified 66,727 FTICR-derived molecular formulas into SOM compound classes, based on thresholds of O:C and H:C ratios in the van Krevelen diagram^90^. The presence/absence matrix of the molecules across the 47 samples were used, to first determine the distribution of SOM types across the four soil sample types, and then to conduct a series of pairwise G-tests of uniqueness^91^ with unadjusted *p* value threshold of 0.05, generating profiles of SOM that are differentially present in one soil type versus the other. The results of the G-tests are shown in Fig. 2. Statistical analyses were conducted in the R package ‘ftmsRanalysis’ ref. ^92^.

### DNA extraction and sequencing

DNA was extracted using *Quick*-DNA Fecal/Soil Microbe Miniprep Kit D6010 (ZymoResearch) and then cleaned and concentrated with DNA Clean and Concentrator-25 (ZymoResearch). Samples were sequenced at Azenta Life Sciences using the Illumina Whole Genome Metagenomics pipeline or at the Joint Genome Institute (JGI), using the Illumina NovaSeq platform (Illumina, Inc., San Diego, CA). The DNA sequences were processed using the JGI's Metagenome Workflow to remove contamination and trim reads that contained an adapter sequence^93^.

### Metagenomic sequence processing

Metagenome construction, including quality-control/read filtering, contig assembly and binning processes, were performed using a suite of tools available in the MetaGEM pipeline^94^. Specifically, short read quality filtering and adapter trimming were applied to the set of reads (de-interleaved) using fastp v0.20.0^95^ with default settings. The filtered reads from each sample were assembled into contigs using MEGAHIT v1.2.9^96^, with *–presets meta-sensitive* and *–min-contig-len 1000* flags.

Before the binning process, contig coverages across the samples (required by some of the binning tools as described below) were generated by cross-mapping the filtered short reads to the set of assemblies. This was accomplished by generating an index for each contig using the “bwa index” command with default settings. The set of indices were used as part of the input for bwa-mem v0.7.17^97^, generating SAM files, which were subsequently converted to BAM format using the “samtools view” command with the *-Sb* flag, and then were sorted using the “samtools sort” command with the default settings. The sorted BAM files were then used to generate the contig coverages for the binning tools, MetaBAT2 v.2.12.1^98^ and MaxBin2 v2.2.5^99^, using the script, “jgi_summarize_bam_contig_depths” available as part of the MetaBAT2 package. The contig coverage files were then used as input data for the MetaBAT2 and MaxBin2, with default settings for each tool.

Binning was performed using the tool CONCOCT v1.1.0^100^ and the scripts available in the MetaGEM pipeline. The coverage files were generated using the script, “concoct_coverage_table.py” (default settings), with a set of BED files generated for each contig as input. The BED files were created using the “cut_up_fasta.py” script, with the parameters *-c 10000 -o 0 -m b* that split the contigs into 10 kb fragments. For each fragment, CONCOCT was run using the coverage file as input and with the *-c 800* parameter, yielding a set of bins for each sample, based on the split contigs. The script ‘extract_fasta_bins.py’ was then run to generate bins using the original unfragmented contigs.

### Assembly of bacterial MAGs and fungal contigs

To extract the best version of bins from the outputs of the three binning tools, the “bin_refinement” script available in metaWRAP v1.2.3^101^ was used with *-x 10 -c 50* parameters. This command de-replicates the results of the three binning methods by selecting the bin with the highest CheckM2^102^ completeness and the lowest contamination, with the completeness taking precedence over contamination. The parameters in this script ensure that only the binning results with contamination and completeness thresholds of ≤ 10% and ≥ 50%, respectively, are considered for the bin refinement process.

To improve the quality of the bins selected from the refinement step, the “reassemble_bins” command (with the same parameters used for the “bin_refinement” script) was used. This script extracts the reads that map to the contigs belonging to the refined bins and reassembles them using the SPAdes assembly. This process is done at both a “strict” (no mismatches) or “permissive” (< 5 mismatches) level. CheckM2 completeness and contamination between the three versions of bins (i.e., strict, permissive or original derived from the refinement) are compared, and the bins with the best score are selected as the finalized MAGs, yielding 344 genomes.

To determine the distribution of the bacterial and archaeal communities that were represented by the MAGs, we first used the “pipe” subcommand in SingleM^103^, generating a summary of operational taxonomic units (OTU) detected based on 35 bacterial and 37 archaeal single-copy marker genes identified from MAGs. The OTU summary table was used as input for the “appraise” function in SingleM, employing the parameters *–imperfect* and *–sequence-identity 0.87* to account for genomes that are similar to those found in the metagenome that were classified at the genus level (i.e. average nucleotide identity of 87%)^104^.

According to the SingleM appraise result, the 344 MAGs represented 44.4% and 32.3% of the bacterial and archaeal diversity. Thus, we enhanced  MAG recovery through coassembly of reads, as implemented in Bin Chicken^83^. Specifically, we used Bin Chicken to identify MAGs through co-assembly of the reads from soils associated with each of the four depth- and respiration-level classification. We first executed Bin Chicken with the single subcommand to generate MAGs from each sample, using forward and reverse reads and SingleM-generated files (e.g., protein-coding genes and two OTU tables—one from contigs and one from MAGs) as input, with the parameters *–run-aviary* and *–assembly-strategy* megahit. The output MAGs from the “single” subcommand were used to execute two rounds of iterative coassemblies via the “iterate” subcommand. For each run of the “iterate” function, we used the same set of parameters as we did with the “single” subcommand. By the end of the second execution of “iterate,” the total number of MAGs increased to 828, improving the SingleM appraisal value to 50.6% and 36.5% for Bacteria and Archaea. The 828 MAGs were dereplicated using galah (v 0.4.0)^105^ at 95% average nucleotide identity similarity, which can be used to represent species-level similarities^106^, resulting in 358 clusters. Representative, dereplicated MAGs from each cluster were selected based on quality metrics from CheckM2. To calculate the abundance of dereplicated MAGs in each sample, filtered reads were mapped to the dereplicated MAG dataset using CoverM^107^ with the following flags: *-p minimap2-sr -m trimmed_mean -min-read-percent-identity 0.80 -min-read-aligned-percent 0.75 -min-covered-fraction 20*. Read mapping indicated that the MAGs accounted for 1.3% to 22.2% of reads from each sample.

The dereplicated MAGs were then taxonomically classified using the “identify”, “align”, and “classify” commands available in GTDB-Tk v2.5.2^108^. This set of commands identifies 120 ubiquitous bacterial marker genes (and 53 archaeal genes) from the input MAGs, which are then concatenated and aligned together. The alignment of the concatenated marker genes is then used as input for Pplacer^109^ to assign each MAG into the optimal loci in the bacterial or archaeal tree of life. The output includes tabular files containing taxonomic classifications of the MAGs. For each depth and respiration-level classification, the average abundances of the taxonomic groups were calculated from the total abundances of the dereplicated MAGs assigned to each lineage.

Finally, we used Kraken2^22^ to recruit DNA reads against fungal taxonomic marker genes present in the RefSeq Fungi^110^ and 18S SSU rRNA sequences from the SILVA database^111^. On average, 1.33 × 10^5^ fungal reads were recovered, making up <1% (i.e., 0.24%) of the total reads from each soil. The reads were assembled using MEGAHIT v1.2.9 with parameters *–presets meta-sensitive –min-contig-len 1000*, generating an average of 86 contigs per sample. The presence of the carbohydrate-active enzymes (CAZymes) in the fungal contigs was assessed by searching them against the Carbohydrate Active Enzymes (CAZy) database^29^, using dbCAN2^112^.

### KEGG pathway analysis of FTICR-derived molecules using KO identifications

The ‘ftmsRanalysis’ package and associated custom scripts were used to generate a summary of KO identifications that mapped to FTICR-derived molecules. Summary tables were parsed to extract KO present in 50% or more of samples belonging to each partition. KEGG pathway representation analysis, which employs the ‘enrichKEGG’ algorithm in the ‘clusterProfiler’ R package^113^, was conducted using this set of KO. This method calculates the ratio of KO affiliated with specific pathways versus the total number of KO in each sample set. This analysis generated a summary visualization of significant KEGG pathways present in each layer/respiration partition (Fig. 4).

### Network analysis of the relationship between the functional potential of MAGs (KO) and FTICR-derived molecules

For each of the four layer/respiration partitions, co-occurrence networks were constructed using Spearman’s correlation to explore the relationship between KO detected from MAGs and FTICR-derived compounds. To generate KO abundance profiles, functional genes were first detected from the nucleotide sequences of all dereplicated MAGs using Prokka^114^ and then searched against KEGG Orthology^115^ using the “annotate” function implemented in EnrichM^116^. For bacterial MAGs, Prokka was executed with *–kingdom Bacteria* and *–addgenes* parameters, while for archaeal MAGs, the *–kingdom Archaea* flag was used instead. Abundance tables of KO and SOM compounds were used as input for Spearman correlation implemented in R. Each correlation matrix was filtered to obtain relationships with *rho* > 0.6 and FDR-corrected *P* < 0.01^117^. The output matrices were then imported into Cytoscape^118^ version 3.10.3. Clusters in each partition were identified using the MCODE application, with default parameters, producing a set of clusters with scores representing their densities and interconnectedness^119^.

### Assessment of microbial carbohydrate depolymerization in abundant prokaryotes using CAZy annotation of MAGs

We selected two bacterial orders, *Rhizobiales* and *Chthoniobacterales*, each of which is represented by >5.0% of the 319 bacterial MAGs recovered from all soil samples. Only one archaeal order (i.e., *Nitrososphaerales*) was selected, as > 90% of the 39 archaeal MAGs were assigned to this taxon. Functional genes detected from MAGs of the selected bacterial and archaeal clades were searched against the Carbohydrate-Active Enzymes (CAZy) database^29^, using dbCAN2^112^ to predict their capacity to depolymerize lignin and other chemically-recalcitrant carbohydrates.

### Metabolic model prediction for *Rhizobiales*, *Chthoniobacterales* and *Nitrososphaerales*

We inferred constructed metabolic model s for the two dominant bacterial orders, *Rhizobiales* and *Chthoniobacterales*, and the archaeal order *Nitrosophaerales *using the functional annotation profiles from above. To accomplish this, we built a metabolic prediction narrative using a selected suite of applications available in the DOE Systems Biology Knowledgebase (KBase)^120^ platform. In this narrative, GFF tables generated using Prokka containing genomic feature information (i.e., gene names, enzyme commission identifications and protein names; see above section) for each detected coding region and the nucleotide FASTA files of the MAGs are used to re-annotate the genomes using RAST-Tk v1.73 (Rapid Annotation using Subsystems Technology - Toolkit)^121^. This process generates the output files compatible with the KBase metabolic modeling applications.

The RAST-Tk annotation objects were used as input for the ModelSEED^122^ application, generating models representing the broader metabolic properties in the bacterial and archaeal lineages under investigation. The output generated by ModelSEED includes a matrix of metabolic reactions, their associated biochemical compounds, and gene-protein-reactions that encompass the dependencies between related genes. Gene-protein-reactions can help differentiate between cases where a reaction is catalyzed by a protein complex encoded by multiple genes and where several individual proteins independently orchestrate a reaction. The ModelSEED output also includes a ‘biomass objective function’ file that indicates the abundance of the metabolites generated by microorganisms as by-products of their growth metabolism.

Finally, the FBA^123^ application was used to read the ModelSEED-derived metabolic models and a default media file provided by KBase, which specifies a set of >500 compounds an organism can use for its growth, ultimately generating tables of estimated growth rates and/or specific metabolites used/produced. FBA estimates the growth rate of an organism by calculating growth-optimal fluxes through all reactions in the metabolic network. The uptake values for the compounds specified in the output file, measured in mmol per gram cell dry weight per hour, indicate whether the microbes use the metabolites for growth or excrete them as a by-product. The list of the output uptake values was examined to determine the spectrum of compounds likely to be metabolized by different microorganisms.

### Reporting summary

Further information on research design is available in the Nature Portfolio Reporting Summary linked to this article.

## Supplementary information

Supplementary Information

Description of Additional Supplementary Files

Supplementary Data

Reporting Summary

Transparent Peer Review file

## Source data

Source Data

## Data Availability

The sequencing data associated with this study are available in the NCBI Sequence Read Archive (SRA) under accession number PRJNA1260013. Data in the MONet open science database can be found at https://sc-data.emsl.pnnl.gov/monet and MONet Zenodo (10.5281/zenodo.7406532). All the raw FTICR-MS data (including SRFA QC samples and blanks) can be downloaded from EMSL data portal (Project ID 60141: https://sc-data.emsl.pnnl.gov/?projectId=60141). The CoreMS processed data can be obtained from Zenodo (https://zenodo.org/records/15328215). The source data for generating Figs. 1–6 can be found in Source_Data.xls. Supplementary Data can be found in Supplementary_Data.xls. Sample metadata and associated metagenomics and natural organic matter data are available through the National Microbiome Data Collaborative (https://data.microbiomedata.org/details/study/nmdc:sty-11-28tm5d36). Source data are provided with this paper.

## Code availability

The CoreMS script and specified parameters used to process the raw FTICR-MS data are available on GitHub (https://github.com/osumpcheng/FTICR_preprocess^124^;). The R codes used for analyzing the FTICR-MS and metagenome data and preparing the main and Supplementary Figs. are publicly available on GitHub (https://github.com/EMSL-MONet/The-1000-Soil-ICR-Metagenome^125^;). The KBase narrative developed to analyze the *Rhizobiales* and *Chthoniobacterales* MAGs can be accessed at 10.25982/3020148.
