# Supplementary material for: Continental-scale integration of soil metagenomes and organic matter chemistry reveals ubiquitous microbial capacity for chemically-recalcitrant carbon decomposition
Source: Nat Commun. 2026 Jun 15;17:5290. doi: 10.1038/s41467-026-71453-5 (PMC13270027; doi:10.1038/s41467-026-71453-5)
Supplement: Supplementary file 1 — Supplementary Information [file 41467_2026_71453_MOESM1_ESM.pdf]

Supplementary Information for “Continental-scale integration of soil metagenomes and organic matter chemistry reveals ubiquitous microbial capacity for chemically-recalcitrant carbon decomposition”

Young C. Song, Cheng Shi, Kelly G. Stratton, Christian Ayala-Ortiz[1], Izabel Stohel, Viviana Freire-Zapata, Malak M. Tfaily, Emiley Eloe-Fadrosh and Emily B. Graham

Supplementary Method

Fourier Transform Ion Cyclotron Resonance Mass Spectrometry (FTICR-MS)

One lab blank and one Suwannee River Fulvic Acid (SRFA) sample (20 ppm) were tested in between every 30 samples (randomized triplicate samples for both surface soil and subsoil at each site) to evaluate instrument performance.

For raw data preprocessing, noise thresholding was performed with signal-to-noise threshold (5 std.), mass error (0.3 ppm), and stoichiometric limits from domain knowledge (C: 1-90, H: 4-200, O:1-23, N: 0-3, S: 0-2, P: 0-1). SRFA standards were used to set a calibration threshold for all soils in the same batch.

Supplementary Figures

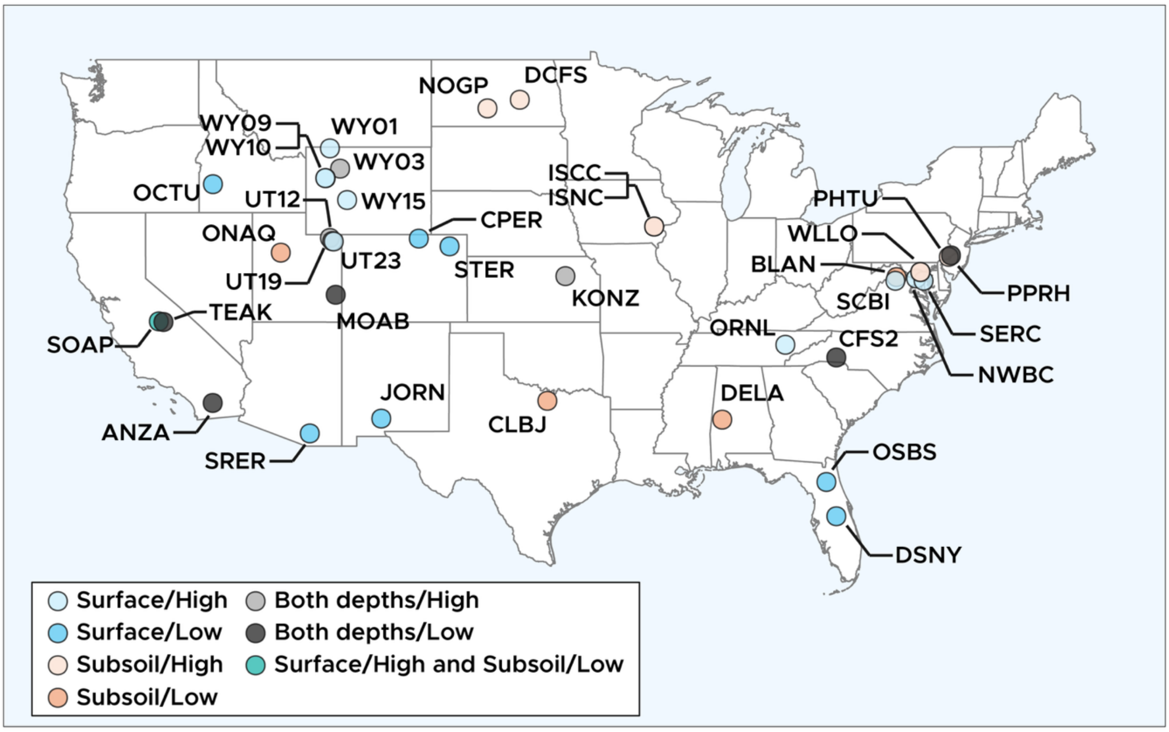

Supplementary Figure 1. Geographic coordinates of 47 soil samples spanning 37 sites across the continental United States (CONUS). Sample types, defined by both soil depths and respiration levels, are represented by color codes as specified in the legend. The map and geographic coordinates were generated using the rnaturalearth, ggplot2, and dplyr R packages. Detailed information of each sampling site, including longitude and latitude are available in Supplementary Table 1.

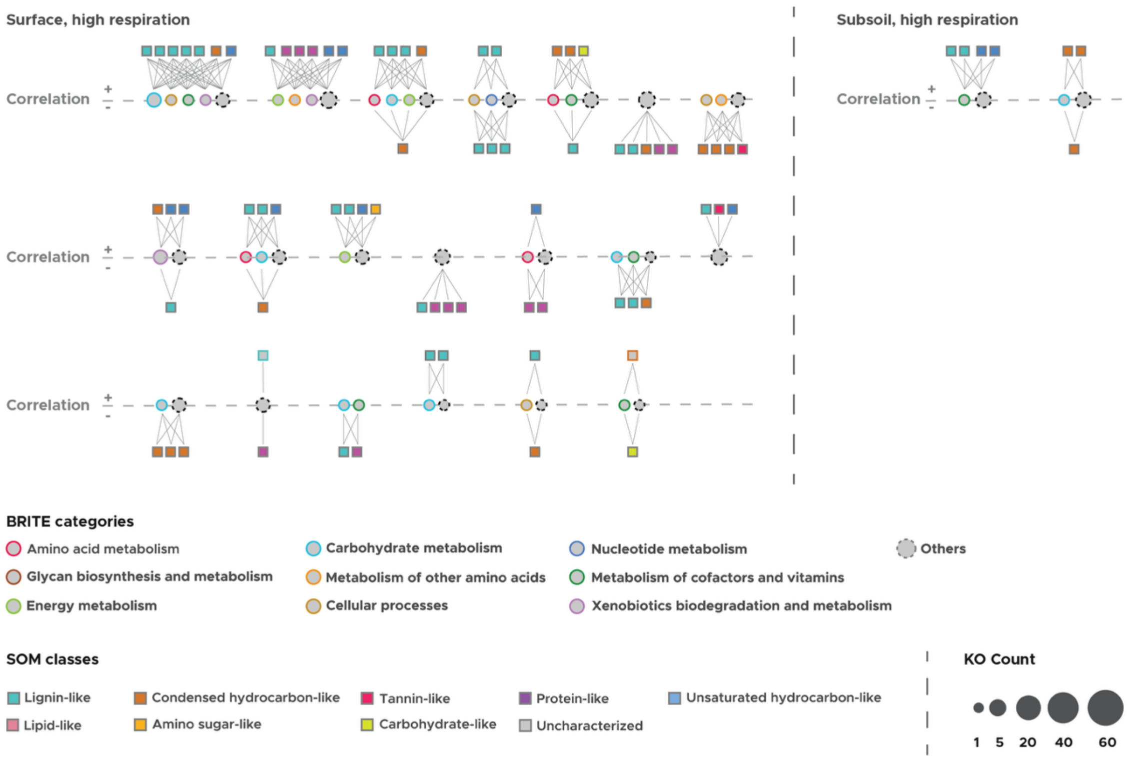

Supplementary Figure 2. Modules with significant correlations between SOMs and KEGG Orthology (KO)-annotated genes detected in high-respiration soils, with the MCODE cluster score of < 5. The shapes of the nodes within each module signify either KOs or SOMs, with their colors representing KEGG pathway categories for KOs or SOM classes, as detailed in the legend. Spearman correlations between SOMs and KOs were calculated, and a two-sided Spearman's rank correlation coefficient test was performed. The figures depicts positive and negative Spearman correlations which had FDR-corrected p-value < 0.01 and |rho| > 0.6, where positive correlations are situated above the dashed line and negative correlations below the dashed line.

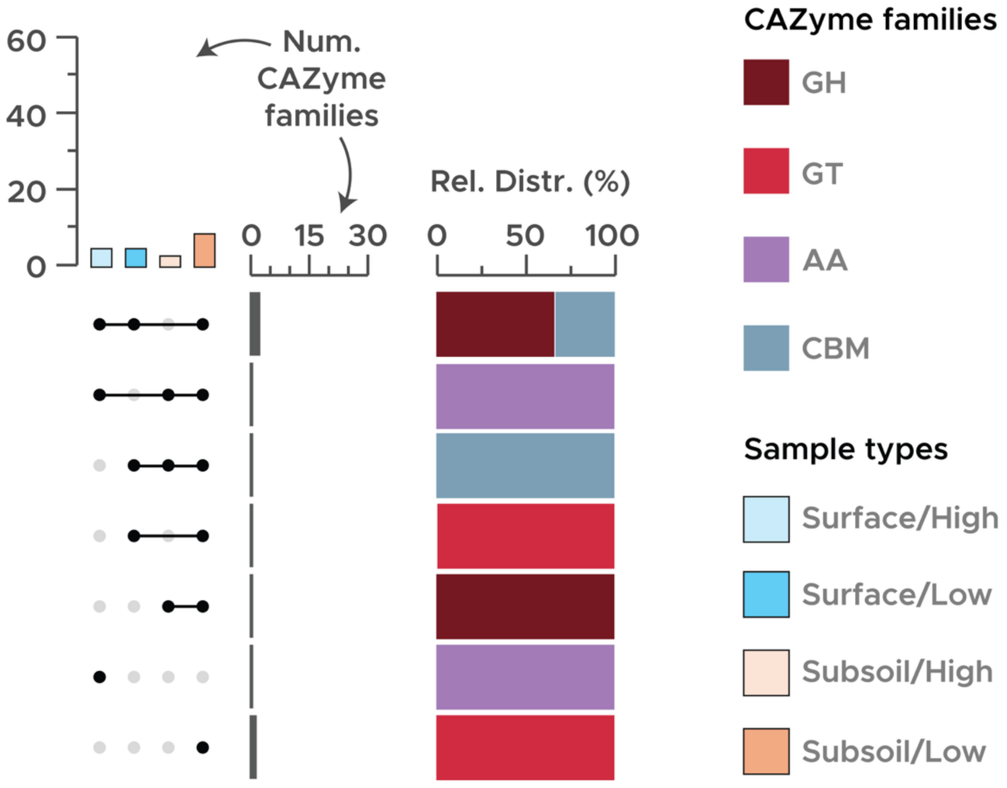

Supplementary Figure 3. Carbohydrate-active enzymes (CAZymes) detected in fungal contigs. The bar chart on the top illustrates the total number of CAZyme families identified from the contigs derived from the surface or subsoils under high or low respiration. Meanwhile, the combined dot-and-line plot along with the bar chart on the left displays the count of CAZyme families found in one or more of the soil sample types. Additionally, the stacked bar chart highlights the proportional distribution of CAZyme categories among these families. The CAZyme categories, as defined in the legend, include GH (glycoside hydrolase), GT (glycosyl transferase), CE (carbohydrate esterase), AA (auxiliary activity), CBM (carbohydrate-binding module), and PL (polysaccharide lyase).
