# Supplementary material for: Continental-scale integration of soil metagenomes and organic matter chemistry reveals ubiquitous microbial capacity for chemically-recalcitrant carbon decomposition
Source: Nat Commun. 2026 Jun 15;17:5290. doi: 10.1038/s41467-026-71453-5 (PMC13270027; doi:10.1038/s41467-026-71453-5)
Supplement: Supplementary file 2 — Description of Additional Supplementary Files [file 41467_2026_71453_MOESM2_ESM.pdf]

Description of Additional Supplementary Files for “Continental-scale integration of soil metagenomes and organic matter chemistry reveals ubiquitous microbial capacity for chemically-recalcitrant carbon decomposition”

Young C. Song, Cheng Shi, Kelly G. Stratton, Christian Ayala-Ortiz[1], Izabel Stohel, Viviana Freire-Zapata, Malak M. Tfaily, Emiley Eloe-Fadrosh and Emily B. Graham

Supplementary Data

Note: Supplementary Data 1-6 are available in Supplementary_Data.xls.

Supplementary Data 1. Geographical coordinates and geophysical properties of 47 soils representing 37 locations, their respiration measurements and DNA (prokaryotic and fungal) read profiles, as indicated by SingleM and Kraken outputs.

Supplementary Data 2. Quality metrics and taxonomic assignments of 358 dereplicated metagenome-assembled genomes (MAGs). 828 MAGs from the 47 soils generated using co-assembly and co-binning processes were dereplicated using galah (v0.4.0) at 95% average nucleotide identity (ANI) similarity. Taxonomic classification of the MAGs, as shown in this table were conducted using the Genome Taxonomy Database Toolkit (GTDBtk).

Supplementary Data 3. Pairwise G-tests of uniqueness between soil types and the molecular compositions of SOM unique to each soil type. The table is divided into six panels, each corresponding to a type of pairwise comparison between soil depths and respiration levels. For each panel, unadjusted p-values are reported for one or both soil types being compared. A gray column indicates that chemistries unique to the designated soil type were not detected, while values in the sample-affiliated columns (i.e., columns other than “Both”) indicate detection of SOM molecules unique to that soil type. The comparisons shown in this table (and in Figure 2) are: Surface/High vs. Surface/Low; Surface/High vs. Subsoil/High; Surface/High vs. Subsoil/Low; Surface/Low vs. Subsoil/High; Surface/Low vs. Subsoil/Low; and Subsoil/High vs. Subsoil/Low.

Supplementary Data 4. List of significant clusters generated using the MCODE method. The “cluster_number” column denotes a module composed of genes (represented by the “ko,” “pathway,” and “gene_name” columns; circles in Figure 3) and SOM chemistry (represented by the “compound” and “som_class” columns; squares in Figure 3) that are connected by edges (represented by the “core” column). The “corr,” “pval,” and “fdr” columns represent the Spearman correlation, unadjusted p-value, and FDR-corrected p-value, respectively, from a two-sided Spearman’s rank correlation coefficient test.

Supplementary Data 5. Raw abundances of KOs associated with amino acid and nitrogen cycle pathways detected in selected genera within the orders *Rhizobiales*, *Chthoniobacterales*, and *Nitrososphaerales*. The columns represent MAGs affiliated with one of the eight genera featured in Figure 6, while rows highlighted in light red indicate genes involved in the reactions visualized in the cell diagram in the same figure. Abundances are based on MAG annotations generated using EnrichM.

Supplementary Data 6. Raw abundances of KOs associated with lipopolysaccharide biosynthesis detected in the selected genera within the order *Rhizobiales*, *Chthoniobacterales* and *Nitrososphaerales*. Abundances are based on MAG annotations generated using EnrichM.
