# Supplementary material for: Continental-scale integration of soil metagenomes and organic matter chemistry reveals ubiquitous microbial capacity for chemically-recalcitrant carbon decomposition
Source: Nat Commun. 2026 Jun 15;17:5290. doi: 10.1038/s41467-026-71453-5 (PMC13270027; doi:10.1038/s41467-026-71453-5)
Supplement: Supplementary file 4 — Reporting Summary [file 41467_2026_71453_MOESM4_ESM.pdf]

Corresponding author(s): Emily B. Graham

Last updated by author(s): Feb 24, 2026

Reporting Summary

Nature Portfolio wishes to improve the reproducibility of the work that we publish. This form provides structure for consistency and transparency in reporting. For further information on Nature Portfolio policies, see our Editorial Policies and the Editorial Policy Checklist.

Statistics

For all statistical analyses, confirm that the following items are present in the figure legend, table legend, main text, or Methods section.

n/a	Confirmed	
☐	☒	The exact sample size (*n*) for each experimental group/condition, given as a discrete number and unit of measurement
☐	☒	A statement on whether measurements were taken from distinct samples or whether the same sample was measured repeatedly
☐	☒	The statistical test(s) used AND whether they are one- or two-sided<br>*Only common tests should be described solely by name; describe more complex techniques in the Methods section.*
☒	☐	A description of all covariates tested
☒	☐	A description of any assumptions or corrections, such as tests of normality and adjustment for multiple comparisons
☐	☒	A full description of the statistical parameters including central tendency (e.g. means) or other basic estimates (e.g. regression coefficient) AND variation (e.g. standard deviation) or associated estimates of uncertainty (e.g. confidence intervals)
☒	☐	For null hypothesis testing, the test statistic (e.g. *F*, *t*, *r*) with confidence intervals, effect sizes, degrees of freedom and *P* value noted<br>*Give P values as exact values whenever suitable.*
☒	☐	For Bayesian analysis, information on the choice of priors and Markov chain Monte Carlo settings
☒	☐	For hierarchical and complex designs, identification of the appropriate level for tests and full reporting of outcomes
☐	☒	Estimates of effect sizes (e.g. Cohen's *d*, Pearson's *r*), indicating how they were calculated

Our web collection on statistics for biologists contains articles on many of the points above.

Software and code

Policy information about availability of computer code

Data collection	The bulk of the metagenomic data was sequenced by Azenta Inc. or at the Joint Genome Institute, both using the Illumina whole-genome metagenomics pipeline.
Data analysis	The CoreMS script and specified parameters used to process the ra FTICR-MS data are available un GitHub (https://github.com/EMSL-MONet/FTICR_Processing). The R codes used for analyzing the FTICR-MS and metagenome data and preparing the main and supplementary figures are publicly available on GitHub (https://github.com/EMSL-MONet/The-1000-Soil-ICR-Metagenome). The KBase narrative developed to analyze the Rhizobiales and Chthoniobacterales MAGs can be accessed at https://narrative.kbase.us/narrative/229872.

For manuscripts utilizing custom algorithms or software that are central to the research but not yet described in published literature, software must be made available to editors and reviewers. We strongly encourage code deposition in a community repository (e.g. GitHub). See the Nature Portfolio guidelines for submitting code & software for further information.

Data

Policy information about availability of data

All manuscripts must include a data availability statement. This statement should provide the following information, where applicable:

- Accession codes, unique identifiers, or web links for publicly available datasets
- A description of any restrictions on data availability
- For clinical datasets or third party data, please ensure that the statement adheres to our policy

Supplementary Tables 1-6 are available in Zenodo (https://doi.org/10.5281/zenodo.18733508). The sequencing data associated with this study are available in the

NCBI Sequence Read Archive (SRA) under accession number PRJNA1260013 (https://www.ncbi.nlm.nih.gov/sra/?term=PRJNA1260013). Data in the MONet open science database can be found at https://sc-data.emsl.pnnl.gov/monet and MONet Zenodo DOI (https://doi.org/10.5281/zenodo.7406532).

All the raw FTICR-MS data (including SRFA QC samples and blanks) can be downloaded from EMSL data portal (Project ID 60141: https://sc-data.emsl.pnnl.gov/?projectId=60141). The CoreMS processed data can be obtained from Zenodo (https://zenodo.org/records/15328215).

Research involving human participants, their data, or biological material

Policy information about studies with human participants or human data. See also policy information about sex, gender (identity/presentation), and sexual orientation and race, ethnicity and racism.

Reporting on sex and gender	*Use the terms sex (biological attribute) and gender (shaped by social and cultural circumstances) carefully in order to avoid confusing both terms. Indicate if findings apply to only one sex or gender; describe whether sex and gender were considered in study design; whether sex and/or gender was determined based on self-reporting or assigned and methods used. Provide in the source data disaggregated sex and gender data, where this information has been collected, and if consent has been obtained for sharing of individual-level data; provide overall numbers in this Reporting Summary. Please state if this information has not been collected. Report sex- and gender-based analyses where performed, justify reasons for lack of sex- and gender-based analysis.*
Reporting on race, ethnicity, or other socially relevant groupings	*Please specify the socially constructed or socially relevant categorization variable(s) used in your manuscript and explain why they were used. Please note that such variables should not be used as proxies for other socially constructed/relevant variables (for example, race or ethnicity should not be used as a proxy for socioeconomic status). Provide clear definitions of the relevant terms used, how they were provided (by the participants/respondents, the researchers, or third parties), and the method(s) used to classify people into the different categories (e.g. self-report, census or administrative data, social media data, etc.) Please provide details about how you controlled for confounding variables in your analyses.*
Population characteristics	*Describe the covariate-relevant population characteristics of the human research participants (e.g. age, genotypic information, past and current diagnosis and treatment categories). If you filled out the behavioural & social sciences study design questions and have nothing to add here, write "See above."*
Recruitment	*Describe how participants were recruited. Outline any potential self-selection bias or other biases that may be present and how these are likely to impact results.*
Ethics oversight	*Identify the organization(s) that approved the study protocol.*

Note that full information on the approval of the study protocol must also be provided in the manuscript.

Field-specific reporting

Please select the one below that is the best fit for your research. If you are not sure, read the appropriate sections before making your selection.

☐ Life sciences ☐ Behavioural & social sciences ☒ Ecological, evolutionary & environmental sciences

For a reference copy of the document with all sections, see nature.com/documents/nr-reporting-summary-flat.pdf

Ecological, evolutionary & environmental sciences study design

All studies must disclose on these points even when the disclosure is negative.

Study description	In this study, we leveraged the integration of soil metagenomic data and soil organic matter (SOM) measurements to explore the microbial diversity and metabolic potential associated with the depolymerization of chemically recalcitrant compounds. To do so, we used data from 47 standardized soil cores—selected using microbial respiration rates from 106 soils across the United States—to assemble 0.76 terabases (Tb) of metagenomic data, recover 828 metagenome-assembled genomes (MAGs), and identify 66,727 distinct SOM molecules.
Research sample	The soil samples analyzed in this study represent 37 locations across the United States and exhibit variations in respiration rates within surface and subsoils. See the "Sampling strategy" section below for the rationale behind sample selection.
Sampling strategy	We investigated 106 soils spanning the continental United States, representing 17 of the 20 ecoclimatic domains defined by the National Ecological Observatory Network (NEON). We then selected 47 soil samples that exhibited the highest and lowest 30% of respiration rates within either surface or subsoils for analysis. These soil samples spanned 37 geographical locations and were divided into four groups based on depth and respiration: surface-high (n = 14), surface-low (n = 12), subsoil-high (n = 11), and subsoil-low (n = 10).
Data collection	Soil metagenome samples were processed and collected by Izabel Stohel. Detailed methods of soil sampling can be found in the manuscript by Bowman et al. (2023; https://www.frontiersin.org/journals/soil-science/articles/10.3389/fsoil.2023.1120425/full). As mentioned in the "Data collection" section, the soil metagenomes were sequenced at Azenta Life Sciences or at the Joint Genome Institute (JGI) using the Illumina whole-genome metagenomics pipeline. The SOM composition in the soils was analyzed using Fourier transform ion cyclotron resonance mass spectrometry (FTICR-MS).

Timing and spatial scale	The soils have been sampled during August of 2022 and February of 2023.
Data exclusions	With the exception of narrowing down the soil samples from 106 to 47, no data was excluded from the study.
Reproducibility	The 1000 Soils is a pilot project of MONet (Molecular Observation Network) at the Environmental Molecular Sciences Laboratory. All MONet data were processed through a standardized, reproducible pipeline.
Randomization	Pre-defined treatment groups are non-existent for this study. All samples were processed as they come in through the reproducible pipeline.
Blinding	See the response of the "Randomization" section above.

Did the study involve field work? ☐ Yes ☒ No

Reporting for specific materials, systems and methods

We require information from authors about some types of materials, experimental systems and methods used in many studies. Here, indicate whether each material, system or method listed is relevant to your study. If you are not sure if a list item applies to your research, read the appropriate section before selecting a response.

Materials & experimental systems

n/a	Involved in the study
☒	☐ Antibodies
☒	☐ Eukaryotic cell lines
☒	☐ Palaeontology and archaeology
☒	☐ Animals and other organisms
☒	☐ Clinical data
☒	☐ Dual use research of concern
☒	☐ Plants

Methods

n/a	Involved in the study
☒	☐ ChIP-seq
☒	☐ Flow cytometry
☒	☐ MRI-based neuroimaging

Plants

Seed stocks	*Report on the source of all seed stocks or other plant material used. If applicable, state the seed stock centre and catalogue number. If plant specimens were collected from the field, describe the collection location, date and sampling procedures.*
Novel plant genotypes	*Describe the methods by which all novel plant genotypes were produced. This includes those generated by transgenic approaches, gene editing, chemical/radiation-based mutagenesis and hybridization. For transgenic lines, describe the transformation method, the number of independent lines analyzed and the generation upon which experiments were performed. For gene-edited lines, describe the editor used, the endogenous sequence targeted for editing, the targeting guide RNA sequence (if applicable) and how the editor was applied.*
Authentication	*Describe any authentication procedures for each seed stock used or novel genotype generated. Describe any experiments used to assess the effect of a mutation and, where applicable, how potential secondary effects (e.g. second site T-DNA insertions, mosiacism, off-target gene editing) were examined.*
