# Supplementary material for: Continental-scale integration of soil metagenomes and organic matter chemistry reveals ubiquitous microbial capacity for chemically-recalcitrant carbon decomposition
Source: Nat Commun. 2026 Jun 15;17:5290. doi: 10.1038/s41467-026-71453-5 (PMC13270027; doi:10.1038/s41467-026-71453-5)
Supplement: Supplementary file 5 — Transparent Peer Review file [file 41467_2026_71453_MOESM5_ESM.pdf]

nature portfolio

Peer Review File

Continental-scale integration of soil metagenomes and metabolomes reveals ubiquitous microbial capacity for recalcitrant carbon decomposition

Corresponding Author: Dr Emily Graham

This manuscript has been previously reviewed at another journal. This document only contains information relating to versions considered at Nature Communications.

This file contains all reviewer reports in order by version, followed by all author rebuttals in order by version.

Version 0:

Reviewer comments:

Reviewer #1

(Remarks to the Author)
The manuscript has been improved substantially compared to the version the reviewer has read before. Additional data on MAGs and analyses have been gained.

This methodogical approach combining MAGs functional analysis with DOC metabolomes of SOM is promissing for future applications, and thus, by itself interesting and has scientific merit.

In the current version of the manuscript methodological limitations regarding sampling scheme, using only prokaryotic MAGs, being not capable of analyzing fungi etc. have been clarified and made obvious.

I found most intruiging that the archaeal order MAGS of Nitrososphaerales contained genes of CAZYmes and often no hydroxalmine oxidoreductase genes. This is really interesting, opens a new route of understanding their ubiquitous distribution and role beyond nitrogen cycling processes. The topic is now well discussed.

I agree that the detection and understanding of complementary differences of aminoacid metabolism between dominant genera MAGs is interesting but somemwhat not too relevant to the main story, i.e. capabilities of soil prokaryotes to degrade lipids and and carbohydrate polymers potentially from SOM pool. This section might be still shortened.

Please as in your cover letter state the proportion of MAGs for total Bacteria (50.6%) and Archaea (36.5%) diversity. This important for any reader to balance the findings in regard to available data.

Minor comments
Please use always the the taxonomic terms 'Bacteria' and Archaea' with a capital starting letter.
In 187 "This modul ..." Which module you mean, the latter? Please rephrase.
in the results section several times the genus name 'Methylocenibacter' has been mispelled 'Methyloacenibacter' remove the 'a'.
In 297, pmoACB is NOT involved in ammonia metabolisms since it encodes the particultae methane monooxygenase. This enzyme can only convert ammonia with low substrate affinity. Is the amo-operon meant here?
In313, what do you mean exactly here?
In 322 a conclusion is missing.
In 332-334, Emphazising again the number of MAGs is redundant and a bit trivial. Please remove it here. It is mentioned in the abstract and results section sufficiently.

(Remarks on code availability)

Reviewer #2

(Remarks to the Author)
Authors have addressed my comments and improved the manuscript markedly. However, I suggest a minor revison adding a short Conclusion section where the author should write:
1) the study confirms what it is well established that soil has a broad metabolic capacity. Soon or later everything is degraded. The importance of the study is use of high-resolution molecular characterization of SOM chemistry combined with the examine of prokaryotic metagenome -assembled genomes;
2) summarize the main innovative message from this study differences in metabolism along the depth

(Remarks on code availability)
Authors have addressed my comments and improved the manuscript markedly. However, I suggest a minor revison adding a short Conclusion section where the author should write:
1) the study confirms what it is well established that soil has a broad metabolic capacity. Soon or later everything is degraded. The importance of the study is use of high-resolution molecular characterization of SOM chemistry combined with the examine of prokaryotic metagenome -assembled genomes;
2) summarize the main innovative message from this study differences in metabolism along the depth

Open Access This Peer Review File is licensed under a Creative Commons Attribution 4.0 International License, which permits use, sharing, adaptation, distribution and reproduction in any medium or format, as long as you give appropriate credit to the original author(s) and the source, provide a link to the Creative Commons license, and indicate if changes were made.
In cases where reviewers are anonymous, credit should be given to 'Anonymous Referee' and the source.
The images or other third party material in this Peer Review File are included in the article’s Creative Commons license, unless indicated otherwise in a credit line to the material. If material is not included in the article’s Creative Commons license and your intended use is not permitted by statutory regulation or exceeds the permitted use, you will need to obtain permission directly from the copyright holder.
To view a copy of this license, visit https://creativecommons.org/licenses/by/4.0/

Responses to the reviewers for the manuscript, “Continental-scale integration of soil metagenomes and organic matter chemistry reveals ubiquitous microbial capacity for chemically-recalcitrant carbon decomposition”

We are grateful to you and to the reviewers for taking the time to read and provide constructive feedback on our manuscript. Below, you will find the list of responses to the comments, including the descriptions of the changes applied to the draft.

Reviewer #1

- **I agree that the detection and understanding of complementary differences of amino acid metabolism between dominant genera MAGs is interesting but somewhat not too relevant to the main story, i.e. capabilities of soil prokaryotes to degrade lipids and and carbohydrate polymers potentially from SOM pool. This section might be still shortened.**
  - We believe that the extent to which we have discussed amino acid metabolism is necessary to clearly distinguish the complementary metabolic strategies present in the bacterial and archaeal genera in this study. The amino acid metabolisms present in these microbes are also one of the mechanisms that enable continued turnover of accessible complex SOM in soil, as addressed in lines 399-403. As such, we have not made any changes in this regard.
- **Please as in your cover letter state the proportion of MAGs for total Bacteria (50.6%) and Archaea (36.5%) diversity. This important for any reader to balance the findings in regard to available data.**
  - This information is already available in line 97 of the “Results” section.
- **Please use always the the taxonomic terms 'Bacteria' and Archaea' with a capital starting letter.**
  - We applied the requested changes throughout the document.
- **ln 187 "This modul ..." Which module you mean, the latter? Please rephrase.**
  - The sentence (lines 189-191) has been rephrased to connect the “module” to the same module that is being described in the previous sentence.
- **in the results section several times the genus name 'Methylocenibacter' has been mispelled 'Methyloacenibacter' remove the 'a'.**
  - The requested changes have been applied throughout the results section.
- **ln 297, pmoACB is NOT involved in ammonia metabolisms since it encodes the particultae methane monooxygenase. This enzyme can only convert ammonia with low substrate affinity. Is the amo-operon meant here?**
  - We agree with this comment, and have changed the instances of “pmoACB” with amoCAB (in line 301)

- **ln313, what do you mean exactly here?**
  - We have added the sentences in lines 317-320 to provide the details on "different strategies in the microbial lineages for surviving carbon limitation."
- **ln 322 a conclusion is missing.**
  - A conclusion that elaborates on the observed partial set of lipopolysaccharide synthesis in the *Chloroflexota* genomes has been added to lines 328-330.
- **ln 332-334, Emphazising again the number of MAGs is redundant and a bit trivial. Please remove it here. It is mentioned in the abstract and results section sufficiently.**
  - This change has been applied.

Reviewer #2

- **Authors have addressed my comments and improved the manuscript markedly. However, I suggest a minor revison adding a short Conclusion section where the author should write:**
  1) the study confirms what it is well established that soil has a broad metabolic capacity. Soon or later everything is degraded. The importance of the study is use of high-resolution molecular characterization of SOM chemistry combined with the examine of prokaryotic metagenome -assembled genomes;
  - We have included a sentence in lines 336-340 to address the already-known degradability of soil carbon.
  - The novelty/importance of this study where multi-omics data are leveraged to re-evaluate the interactions between soil carbon and microbial communities is addressed in lines 347-349.

2) summarize the main innovative message from this study differences in metabolism along the depth

  - A summary of main findings has been added to lines 349-352.

Additional change(s)

- We made changes to the "Data Availability" and "Code Availability" statements, including insertion of DOI references for the supplementary tables and the code (both the GitHub repositories and the KBase narrative).
- We updated the "Acknowledgement" statements to include the most recent statement for MONet, and to recognize Zach Crockett at ORNL for the assistance with implementing the KBase narrative.
